# A Modular Analysis of the Auxin Signalling Network

**DOI:** 10.1371/journal.pone.0122231

**Published:** 2015-03-25

**Authors:** Etienne Farcot, Cyril Lavedrine, Teva Vernoux

**Affiliations:** 1 Centre for Mathematical Medicine and Biology & Centre for Plant Integrative Biology, School of Mathematical Sciences, University of Nottingham, Nottingham, UK; 2 Laboratoire de Reproduction et Développement des Plantes, CNRS, INRA, ENS Lyon, UCBL, Université de Lyon, Lyon, France; Centrum Wiskunde & Informatica (CWI) & Netherlands Institute for Systems Biology, NETHERLANDS

## Abstract

Auxin is essential for plant development from embryogenesis onwards. Auxin acts in large part through regulation of transcription. The proteins acting in the signalling pathway regulating transcription downstream of auxin have been identified as well as the interactions between these proteins, thus identifying the topology of this network implicating 54 Auxin Response Factor (ARF) and Aux/IAA (IAA) transcriptional regulators. Here, we study the auxin signalling pathway by means of mathematical modeling at the single cell level. We proceed analytically, by considering the role played by five functional modules into which the auxin pathway can be decomposed: the sequestration of ARF by IAA, the transcriptional repression by IAA, the dimer formation amongst ARFs and IAAs, the feedback loop on IAA and the auxin induced degradation of IAA proteins. Focusing on these modules allows assessing their function within the dynamics of auxin signalling. One key outcome of this analysis is that there are both specific and overlapping functions between all the major modules of the signaling pathway. This suggests a combinatorial function of the modules in optimizing the speed and amplitude of auxin-induced transcription. Our work allows identifying potential functions for homo- and hetero-dimerization of transcriptional regulators, with ARF:IAA, IAA:IAA and ARF:ARF dimerization respectively controlling the amplitude, speed and sensitivity of the response and a synergistic effect of the interaction of IAA with transcriptional repressors on these characteristics of the signaling pathway. Finally, we also suggest experiments which might allow disentangling the structure of the auxin signaling pathway and analysing further its function in plants.

## Introduction

Auxin is a key signal for most of organogenesis and patterning processes occurring during plant development, in both shoot and root. The nature and intensity of the cellular response to this signal is known to be regulated at many levels, among which are the biosynthesis and polar transport of auxin that control the spatio-temporal distribution of the signal, but also the cellular sensitivity to the auxin signal [1]. It is still unclear how these different layers of control are integrated and allow for regulating auxin responses during plant development. In particular, the contribution of the topology of the pathway to the cellular sensitivity to auxin is still largely unknown.

The auxin transduction network is mainly composed of two classes of transcriptional regulators encoded by multigene families, the Auxin Response Factors (ARFs; 23 proteins) and Aux/IAAs (shortened here as IAAs; 29 proteins). ARFs are transcription factors that can be either activators or repressors. In the absence of auxin, activator ARFs (ARF+; 5 proteins) form heterodimers with IAA proteins. It has been proposed that IAA can recruit TOPLESS (TPL)/TOPLESS-RELATED (TPR) co-repressors when the ARF+:IAA heterodimers are bound to the promoters of the target genes [2, 3]. ARF^+^:IAA hetero-dimerization thus also allow for an active repression of target gene transcription. IAAs can also form dimers with themselves while repressor ARFs (ARF-) show very limited interaction with IAAs. ARF- may act mainly by competing with ARF+ on target gene promoters since they have been proposed to target identical sequence motifs in the promoter of auxin-regulated genes [4–6].

**Fig 1:**
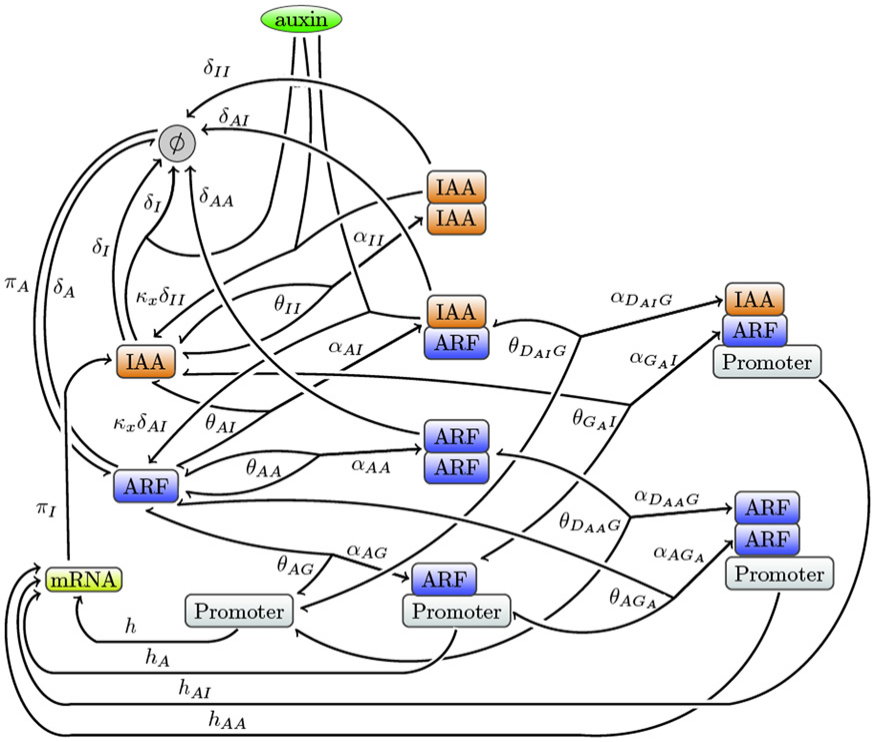
Reaction diagrams for the auxin pathway. Detailed diagram representing all elementary reactions. The used graphical convention is described Material and Methods. Activator or repressor ARFs can be represented using respectively high or low values for both the transcription rates *h*_*A*_ and *h*_*AA*_. The symbol ∅ represents the empty set, introduced as usual to model degradation and constitutive expression of genes.

When present in the cell, auxin can bind to TIR1/AFB F-box nuclear co-receptors, which are part of an SCF E3 ubiquitin ligase complex [7–10]. This leads to the interaction between TIR1/AFBs and IAAs, thus promoting their degradation by the proteasome. ARF^+^ are eventually released and can thus enhance their target gene transcription, leading to the cellular responses (differentiation, division, etc.). An overview of the transduction network is provided in Fig. 1.

In addition to this general scheme, the fact that the ARFs and IAAs are encoded by large multigene families contributes to the signalling capacity of this transduction pathway. The 29 members of the IAA family, the 5 ARF^+^ and the 18 ARF^−^ have different biochemical properties, for instance potentially various interaction affinities and in the case of IAAs a wide range of turn-over rates, hence providing potential quantitative modulation of the auxinic response at the cellular scale depending of the local combination of IAAs and ARFs [1]. The different ARF and IAA show a diversity of expression patterns at different stages of plant development further highlighting the regulatory potential linked to such combinations [11–13]. Also, in support to this idea, it has been shown by expressing dominant-negative form of IAAs and ARFs under identical promoters that these proteins are only partly redundantly functional [14, 15]. In addition, most IAA genes are induced by the auxin signal [16]. Some ARF genes, namely ARF4, 5 and 19 are also positively regulated downstream of auxin possibly in an ARF-dependent fashion [16–18]. These feedbacks confer non-linear transduction capacities to the network. As a consequence, it appears that simple differences in the topology (i.e. the structure of interactions between molecular species) of this transduction network could significantly influence the nature of the response to a given input.

Modelling of the auxin signalling pathway is a powerful way to explore the regulatory potential of this pathway central to plant development and to pinpoint potential properties than can be further analysed experimentally. The inherent complexity of the auxin pathway can then be approached with two complementary viewpoints, which have received many names in the literature. The analytic (or reductionist among other terms) approach considers individual modules whose function contribute to signal transduction. The synthetic (or holistic) viewpoint, on the other hand, assumes as stated in [19] that “the properties of the components depend on the system of which they are part” and studies the pathway as a whole. As both viewpoints are complementary rather than exclusive, the strategy adopted in this paper is to rely on both. Our aim will be to analyse the contribution of different topological elements of the network in the context of this whole pathway, using simple models of these individual modules as a heuristic guide to intuition. More specifically, the first motivation of this study stems from the observation that the auxin pathway includes two distinct paths, or modules, pointed out above and likely contributing together to gene transcription regulation by auxin:

- A *sequestration* module: the formation of ARF:IAA dimers can sequestrate activator ARFs away from promoters and this repressive function is released upon auxin-induced IAA degradation.
- An *active repression* module: once bound to a promoter, ARF:IAA dimers act in conjunction with other regulators such as TPL/TPR to repress transcription and this repression is also released upon auxin-induced IAA degradation.

While active repression through TPL/TPR recruitment has been clearly demonstrated experimentally to play a key role [3], very little is known on the contribution of sequestration. Also the contribution of the formation of the different dimers and feedback regulations, that can also be seen as different regulatory modules are still elusive. As we aim to explore the potential contributions of these modules, we include the minimum required elements to represent them all. This dictates the level of detail of our model: it has to include distinct variables for ARF:IAA dimers in solution and immobilised on a promoter. For consistency, this also applies to IAA:IAA and ARF:ARF dimers. For parsimony, on the other hand, we do not include the details of auxin perception by the TIR1/AFB F-box nuclear co-receptors and simplify this process using quasi-steady state assumptions.

These hypotheses and others of a more technical nature are detailed in the first section, allowing us to define an ordinary differential equations (ODE) model of the auxin pathway. Different modules that compose the model are then described, before defining different characteristic outputs, all interpreted as functions of auxin. This allows to explore the potential influence of these modules on the input/output behaviour of the auxin pathway in the remaining sections using the strategy described above.

## Results

### General hypotheses

The starting point of the model definition consists in establishing a list of considered molecules, and of all the elementary reactions between them. Our model focuses on the signalling cascade that follows the perception of auxin by its AFB/TIR1 receptors, as described in more details below. The molecules we consider are two populations of ARF and IAA proteins respectively, as well as an auxin responsive gene whose promoter can bind with the considered ARF and all ARF bearing dimers. Our model does not address the question of auxin transport, and the spatial resolution of the model is that of a single cell. The influence of larger spatial scales are still accounted for in two ways: first, the concentration of auxin will be considered as an arbitrary input, which could thus be defined differently in different locations in a tissue. Secondly, different tissues will in general correspond to different distributions of ARF and Aux/IAA proteins, which can be reflected in the model by choosing different values of the kinetic rates.

Note that the two protein populations could be interpreted either as a specific pair of ARF and IAA, say ARF5 and IAA12, or as two populations comprising distinct members of the ARF and IAA families, in which case the kinetic constants shall be interpreted as *apparent* constants.

We consider the following reactions:

- **Dimerization:** If it is clearly established that ARF:IAA and IAA:IAA dimers can form [13, 20–23], there was contradictory results concerning ARF:ARF dimers in the literature [13]. While other studies have found such dimers, though often with weaker intensity than ARF:IAA which could explain these discrepancies [21–25]. However recent structural and biochemical studies have clearly demonstrated ARF:ARF interactions in solution through both their DNA binding domain and a specific protein-protein interaction domain [4, 26, 27]. We thus consider that all types of homo and hetero-dimers can form, in a reversible way.
- **Transcriptional regulation:** We consider that ARF proteins and every ARF bearing dimer can be found attached to the promoter. In the case of a dimer, we consider that it can be formed either prior to binding (“dimer pathway”) or from a DNA bound ARF (“monomer pathway”), as both pathways can occur in gene regulation by dimers [28].
  By assigning a specific transcription rate (see below) to each configuration of the promoter resulting from these binding events, we account for the regulation of transcription. This implies that the role additional repressing factors which are known to bind to ARF:IAA on a promoter (notably TPL/TPR) is modelled by these transcription rate parameters.
- **Feedback** We do consider feedback on IAA, in the sense that the target gene codes for the considered IAA protein, with different transcription rates depending on how the promoter is bound to different types of regulatory complexes. To represent this feedback we have to include the mRNA coding for IAA proteins explicitly in the model. Production of ARF proteins, on the other hand, is supposed constant by default as only a few ARFs are known to be regulated (a particular case which considered in a later section).
- **Degradation:** All proteins and mRNAs are supposed to be degraded with a linear rate. Usually, the degradation rates of all proteins or dimers represent both dilution through growth (slow; *τ*_1/2_ ∼ 40–100min) and proteasome mediated degradation (fast; *τ*_1/2_ ∼ 3-30min). Auxin signalling being typically much faster than the first of these two processes, we will in fact ignore effects of growth and dilution, and only consider degradation mediated by the proteasome machinery.
- **Auxin perception:** Since auxin acts by enhancing degradation of IAA proteins by the proteasome, we incorporate this action by making the decay rate of IAA proteins an increasing function of auxin levels. We do not consider explicitly the detailed mechanism of auxin perception, through binding of IAA to the SCF^TIR1/AFB^ complex. However, it was shown in our previous study [13] that a quasi-steady state assumption for auxin perception steps leads to the IAA decay rate being a Michaelis-Menten function of auxin. Here, we assume that the system performs in a regime where auxin levels do not saturate this decay rate, which is thus nearly linear. In consequence the parameter *x* is in fact a parameter combining the concentration of both auxin and the perception complex SCF^TIR1/AFB^. It is not known whether auxin induced degradation affects IAA proteins already bound in a dimer. More generally, dimer levels can decrease through dissociation into monomers or through degradation as dimers. The latter would be more likely if dimers resulted from obligate protein-protein interactions, whereas transient protein-protein interactions would lead to dimer populations decaying predominantly through dissociation. For sake of generality, our default assumption will be that both effects could play a role of comparable intensity, like in [13] but other scenarios are also considered, see Section “Forms of auxin induced degradation”.

Note that we have not specified at this stage whether the considered ARF is an activator or a repressor and in principle both situations could be represented in our model. However, unless specified our default assumption will be to consider activator ARFs. In particular, the assumptions above would need slight alterations for typical repressor ARFs: these proteins are indeed involved in very little dimerization (with either ARF or IAA) see [13], allowing to ignore the first type of reaction above, hence making the discussion about dimers and transcriptional regulation unnecessary.

Based on the assumptions above, a model consisting of 10 ODEs can be defined, as detailed in the *Model* section. This section defines the different parameters involved in the model and their values when known, resulting in the model shown in Equation (5).

### Modular structure

**Fig 2:**
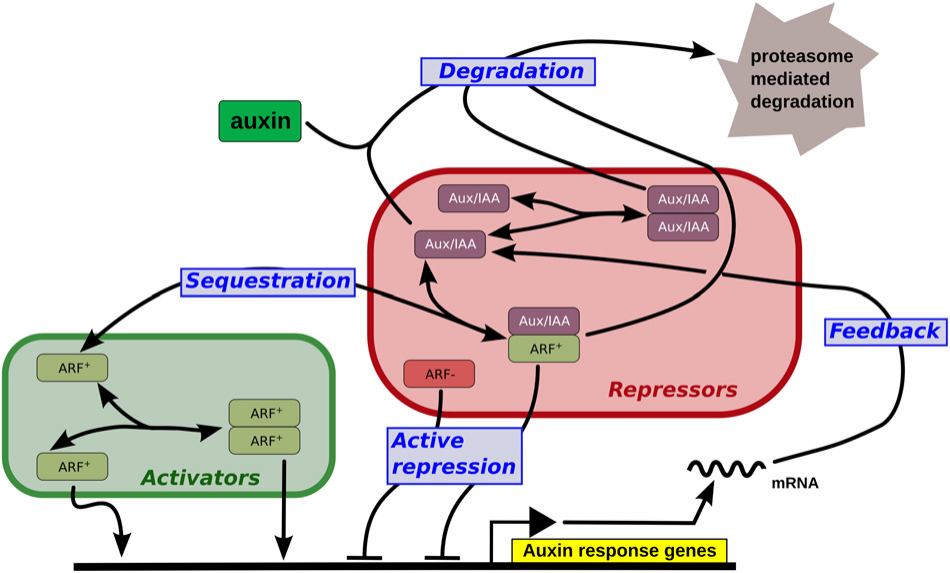
Defining modules. A simplified representation of the auxin pathway, where the different configurations of the promoter and the natural degradation (i.e. not auxin induced) of proteins and dimers are not shown. The five functional modules studied in this paper are shown using blue boxes.

To decipher the modular organization of this network, we will consider some specific sub-networks whose biological function and mathematical representation can be investigated individually. In particular, we will focus in the following subsections on the five modules below, also represented in Fig. 2.

- **Sequestration** of ARF proteins through dimerization with IAA.
- **Active repression** of transcription by ARF:IAA dimers.
- **Dimer formation** among potentially all pairs of IAA or ARF proteins.
- **Feedback** loop due to most IAAs and some ARFs being auxin regulated.
- Auxin induced **degradation** of IAA and potentially of all IAA bearing dimers.

From Fig. 2 notably, it appears that the five processes listed above do not correspond to modules in the sense of sub-networks with a specific topology, like for instance the so-called network motifs [29]. They are less formally defined and correspond to a biological function. From the more detailed reaction scheme shown in Fig. 1 it also becomes apparent that these modules cannot be reduced to a single parameter in the model. Each module is determined by specific parameters from Equation (5) as follows:

- Sequestration is primarily controlled by parameters *α*_*AI*_ and *θ*_*AI*_.
- Active repression is controlled by parameters *α*_*G*_*A*_*I*_, *θ*_*G*_*A*_*I*_, *α*_*D*_*AI*_*G*_, *θ*_*D*_*AI*_*G*_ and *h*_*AI*_.
- Dimer formation is controlled by all pairs of parameters of the form *α*_*XY*_, *θ*_*XY*_.
- Feedback is controlled by parameter *π*_*I*_, and *π*_*A*_ when feedback on ARF is considered in a later section.
- Auxin induced degradation is controlled by *δ*_*I*_, *δ*_*II*_, *δ*_*AI*_ and *κ*_*x*_.

It appears that there is some overlap between these sets of parameters. In the following each module will be discussed separately but these intricacies will not be ignored.

### Input/output interpretation

As a signal transduction pathway, the network modelled in (5) is naturally interpreted as an input/output (I/O) system, which processes an input of auxin level to return an appropriate response. More precisely, the natural input for the system (5) is the dimensionless variable *x*, which can be interpreted as the combined level of auxin and TIR1/AFB co-receptors in the medium, as discussed more formally in [13].

The output, on the other hand could be chosen in several meaningful ways. The most obvious is probably the differential level of transcribed mRNA in response to auxin, both expressed in absolute value or relative to the auxin-free transcription level. However, the auxin signalling pathway is not only known for inducing transcription in response to auxin, but also for doing this very fast (IAAs can be degraded in just a few minutes [30] and the transcriptional response to auxin occurs within minutes [31]). Finally, a meaningful characteristic of every transduction pathways is its relative fold change in response to a fold-change of the input, also called *sensitivity*, or *logarithmic gain*; in particular, it is a known feature of various pathways to present ultrasensitivity, notably in presence of sequestration mechanisms [32, 33], which are present in the auxin pathway (ARF being sequestrated by IAA) and will be discussed in later sections. Therefore, one will consider that the model (5) has a one-dimensional input *x*, and a multi dimensional output comprising typical response intensities, a typical response time and a sensitivity.

Let us now define these outputs more precisely. Let R*(*x*) denote a steady-state value of the mRNA concentration *R* for an auxin level *x*. Note here that if the pathway had several steady states for some parameter values, each input/output function will be understood to be *relative to a steady state*, which will be specified when needed.

The relative increment in response to an increase *x* of the auxin input will be called relative response intensity, denoted

$$\begin{array}{c} \rho_{rel}\left(x\right)=\frac{R^{*}\left(x\right)-R^{*}\left(0\right)}{R^{*}\left(0\right)}. \end{array}$$ (1)

Similarly, the absolute response intensity is defined as

$$\begin{array}{c} \rho_{abs}\left(x\right)=R^{*}\left(x\right)-R^{*}\left(0\right). \end{array}$$ (2)

The sensitivity is given by the different forms below

$$\begin{array}{c} \sigma \left(x\right)=\left(\frac{dR^{*}}{R^{*}}\right)/\left(\frac{dx}{x}\right)=\frac{x}{R^{*}\left(x\right)}\frac{dR^{*}}{dx}\left(x\right)=\frac{d}{dx}\left(\log R^{*}\left(x\right)\right). \end{array}$$ (3)

Finally, the response time will be defined as the minimum time after which the system remains ‘close’ to steady state (bearing in mind that being exactly at steady state takes an infinite time), given an initial condition. This quantity is thus relative to the considered initial condition and also to how close to steady state the solution has to be. In the rest of this paper we will consider differing by less than 5% to be close. One will also consider an initial condition which is equal to the steady state without auxin (with the identically zero initial condition). More formally:

$$\begin{array}{c} \begin{array}{ccc} \tau (x) & = & \inf\left\{t>0\;:\;\;\forall t^{'}>t,\;|R\left(t^{'},x\right)-R^{*}\left(x\right)|<\frac{5}{100}R^{*}\left(x\right)\right\}, \end{array} \end{array}$$ (4)

where *R* is given by solving (5) with the initial condition described above and auxin input *x*, R*(*x*) being the steady state associated to this solution.

Note that, unlike the three other outputs, the response time is expected to take lower values in the more favourable situations.

### Influence of parameters on I/O behaviour

Now that several interpretations of the auxin signalling pathway as an I/O system have been defined, we focus on the influence of parameters on the system. Since an exhaustive approach is made impossible by the high number of parameters, we rely on the modular decomposition described in the previous section and analyze the influence of some key parameters controlling one or several of the five modules. Besides being easier than a comprehensive exploration of the parameter space, it allows for a natural interpretation in biological terms.

#### The core mechanism: sequestration vs. active repression

To begin with, we shall consider the two main mechanisms by which auxin can lead to transcription of its target genes. Indeed, the overall *modus operandi* of the system consists in releasing the ARF from IAA to induce transcription. This de-repression acts at two levels: ARF are released from ARF:IAA dimers, corresponding to our sequestration module, and the decrease of the IAA population also reduces transcriptional repression, corresponding to our active repression module.

As there is no clear evidence in the literature allowing to exclude one of these paths, we will start our investigation by exploring their relative influences on the input/output behaviour of the whole system. To do so, one introduces a new parameter *λ* ∈ [0, 1] such that *λ* = 0 and *λ* = 1 correspond to a situation without sequestration and a situation without active repression, respectively. Intermediate values of *λ* correspond to both mechanisms being operative. Specifically, one replaces in Equation (5):

- all terms *α*_*AI*_ *IA*−*θ*_*AI*_ *D*_*AI*_ by *λ*(*α*_*AI*_ *IA*−*θ*_*AI*_ *D*_*AI*_),
- all terms *α*_*G*_*A*_*I*_ *IG*_*A*_−*θ*_*G*_*A*_*I*_ *G*_*AI*_ by (1−*λ*)(*α*_*G*_*A*_*I*_ *IG*_*A*_−*θ*_*G*_*A*_*I*_ *G*_*AI*_),
- all terms *α*_*D*_*AI*_*G*_ *GD*_*AI*_−*θ*_*D*_*AI*_*G*_ *G*_*AI*_ by (1−*λ*)(*α*_*D*_*AI*_*G*_ *GD*_*AI*_−*θ*_*D*_*AI*_*G*_ *G*_*AI*_).

Then, one wishes to consider the effect of varying *λ* on the different input/output functions (1)–(4). How these functions depend on both *x* and *λ* can be interpreted geometrically as a two dimensional landscape. Since other parameters of the system may in general have a strong effect on its input/output behaviour, we have in fact considered such landscapes for different values of the parameters. Because (5) comprises over 20 parameters, a complete exploration is excluded, even using advanced computational techniques and hardware. Since decay rates are the most well known parameters of the system, they were fixed close to experimentally observed values, as reported in Table 1 and Section “Models”. On the other hand, binding and unbinding rates are not known and could in principle vary by several orders of magnitude. We thus chose to vary these parameters, within plausible ranges based on the literature [32].

**Table 1:** Default parameter values.

δ_*I*_	δ_*A*_	δ_*AA*_	δ_*AI*_	δ_*II*_	δ_*R*_	π_*I*_	π_*A*_	*h*	*h*_*A*_	*h*_*AA*_	*h*_*AI*_	κ_*x*_
0.05	0.003	0.003	0.003	0.003	0.007	10	1	1	10	10	0	10

Reference when no specific values are provided. Other parameters are association constants (denoted *α*_*XY*_) and dissociation constant (denoted *θ*_*XY*_) and are specified on a case by case basis, due to a lack of experimental data regarding these constants. Concentrations are expressed in nM and time in min, see Section “Model” for details of parameter units and the literature used to specify the default parameters.

Even with this restriction the number of parameters to vary was still computationally daunting, leading us to additional simplifying assumptions: we distinguished between protein-protein and protein-promoter binding reactions, but within each two types we assumed that all association (resp. dissociation) rates of the form *α*_*XY*_ (resp. *θ*_*XY*_) were equal.

The parameters *α*_*XY*_ were varied over 5 orders of magnitude. By default, from comparable cases found in the literature they took values (in nM^−1^.min^−1^ for protein-protein association and min^−1^ for protein-promoter association) in the sample set

$$\begin{array}{c} S_{\alpha }=\left\{0.001,\;0.01,\;0.1,\;1.0,\;10.0\right\}, \end{array}$$

whereas the parameters *θ*_*XY*_ were varied over 6 orders of magnitude, taking default values (in min^−1^ for protein-protein association and nM.min^−1^ for protein-promoter association) in

$$\begin{array}{c} S_{\theta }=\left\{0,\;0.001,\;0.01,\;0.1,\;1.0,\;10.0\right\}. \end{array}$$

The value 0 was not included for *α*_*XY*_ because it corresponds to an extreme situation where no dimers can form, which leads to an absence of response to any auxin input. Even with the assumptions made at this point, exploring all combinations involves 900 cases, which would have led to an unreasonably long computational time. We thus restricted the exploration by imposing that protein-protein and protein-promoter constants vary by at most one order of magnitude.

We use a notational convention to describe all the considered cases. Denote *α*_*P*_ < *α*_*AG*_ a situation where the association constants for protein-promoter vary in *S*_*α*_ and association constants are one order of magnitude smaller, hence varying in *S*_*α*_/10 = {0.0001, 0.001, 0.01, 0.1, 1.0}. Similarly, we denote *θ*_*P*_ < *θ*_*AG*_ when the dissociation constants for protein-protein binding reaction are one order of magnitude smaller than constants for protein-promoter dissociation (and thus vary in *S*_*θ*_/10 instead of *S*_*θ*_). Table 2 summarizes all the cases we have explored, with the natural generalization of the notation just described:

**Table 2:** Additional parameter sets.

	*α*_*P*_ = *α*_*AG*_	*α*_*AG*_ < *α*_*P*_	*α*_*P*_ < *α*_*AG*_
*θ*_*P*_ = *θ*_*AG*_	“default”	A	B
*θ*_*AG*_ < *θ*_*P*_	C	E	G
*θ*_*P*_ < *θ*_*AG*_	D	F	H

Since for each case the ratio between protein-protein and protein-promoter constants was fixed, this left us with two global parameters *α* and *θ* that were varied in *S*_*α*_ and *S*_*θ*_ respectively, used as ranges for the higher constants in cases where they differed between protein-protein and protein-promoter.

To chose a relevant range of values for *x*, we performed a preliminary exploration to ensure that a non-negligible response was obtained in all or most explored parameter regimes. This led us to empirically define the range *x* ∈ [0, 2000].

**Fig 3:**
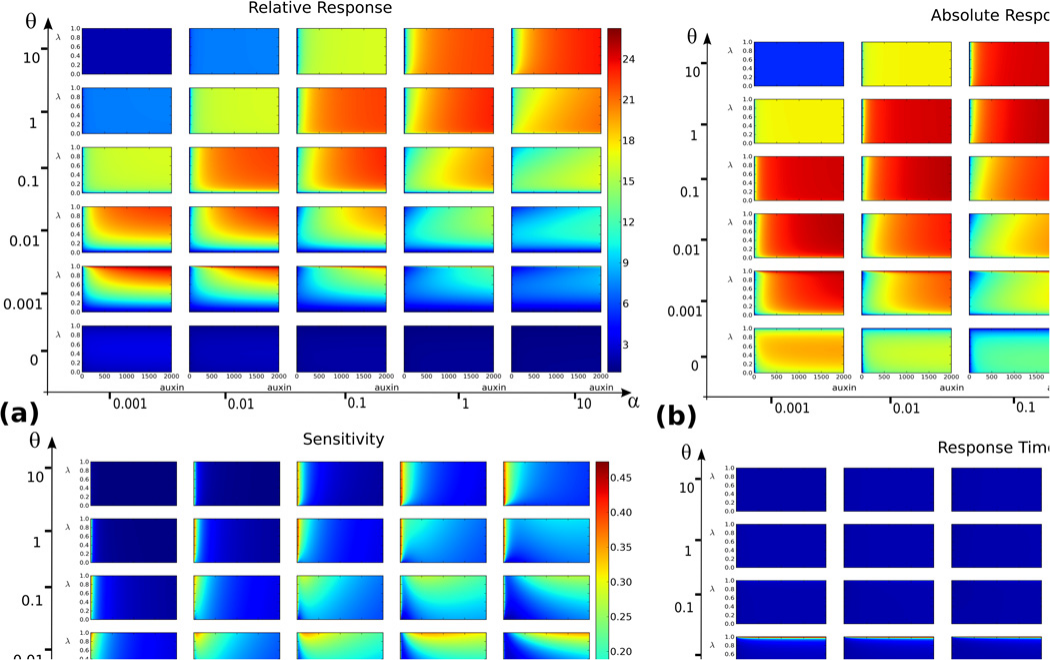
Output landscapes as functions of auxin level *x* (abscissae) and balance between the two core mechanisms, parametrized by *λ* (ordinates). **(a)** Relative response *ρ*_*rel*_(*x*,*λ*). **(b)** Absolute response *ρ*_*abs*_(*x*,*λ*). **(c)** Sensitivity *σ*(*x*,*λ*). **(d)** Response time *τ*(*x*,*λ*). For each of these functions, the grid of values (*α*,*θ*) ∈ {0.001,0.01,0.1,1,10}×{0,0.001,0.01,0.1,1,10} is considered and for each landscape (*x*,*λ*) span a 200×200 regular grid on the rectangle [0, 2000]×[0, 1].

In summary, for each of the 9 cases above we computed numerically the values of the 4 functions (1)–(4) for a 200×200 regular grid on the domain (*x*,*λ*) ∈ [0, 2000]×[0, 1]. This operation was repeated for a coarse grid of values taken by the two global parameters *α* and *θ*. The complete computation was distributed on 8 cores (2 cores per machine on 4 recent stations having between 6Gb and 24Gb memory per core) and took over 597h ≈ 24.88 days to be complete. The results for the default situation, “*α*_*P*_ = *α*_*AG*_” & “*θ*_*P*_ = *θ*_*AG*_” are shown in Figs. 3. Remarkably, the corresponding figures of the 8 other cases A-H (S1 Additional Information), did not differ significantly from the default parameter choice, whose main features are discussed below.

The landscapes shown on Fig. 3 present several features. First at the coarse grid level it appears that except for the response time, the output functions are far from uniform on the coarse grid: some regions in the (*α*,*θ*) space are more favourable than others. Both the absolute and relative responses reach higher values for $\frac{\theta }{100}\leq \alpha \leq \theta$, corresponding to a dissociation constant $K_{d}≐\frac{\theta }{\alpha }$ in the range [1nM, 100nM], i.e. strong to moderately strong dimers [32]. Within this range, the landscapes corresponding to *K*_*d*_ = 100 in fact have a globally high absolute response but a lower relative response than for more stable dimers. Along the *x* axis, higher values of the output functions are increasing, reaching a maximal value at a critical auxin level after which they tend to plateau.

In the range *K*_*d*_ ∈ [1nM, 10nM] of stronger dimers notably, there seems to be a minimal *λ* value below which *ρ*_*rel*_ landscapes stay low and never reach their plateau. For *θ* ≤ 0.01 for instance, i.e. the 3 lower rows in Fig. 3(a), there clearly seems to be a restricted range of *λ* values close to *λ* = 1 leading to higher responses. Though less striking, this phenomenon also appears on *ρ*_*abs*_ landscapes, where one also notices (e.g. on the row *θ* = 0.001) a value of *λ* below which no plateau is reached.

Although the ranges of *K*_*d*_ values changes from the above, the qualitative features of the default parameter choice were found to be conserved for the cases A-H. This indicates that variations within the association and dissociation rates may have a tuning effect on the values at which the different outputs are maximal, but do not affect the following general feature: the relative and absolute response landscapes present an optimum plateau, which lies above a minimal value of *λ* > 0 and does not include *λ* = 1.

In the case of the sensitivity *σ*, there appears to be a specific and narrow range of *λ* and *x* values at which sensitivity is maximal, Fig. 3(c). This specific range is dependent on the (*α*,*θ*) values on the coarse grid, a fact reminiscent of the role of a combinatorics of dimers (and thus dimerization kinetic constants) as a means to modulate auxin response [7]. This fact will be discussed further in later sections. In the present case, it is also important to notice that the maximal values of *σ* reached on all grids do not exceed 1. In other words, for none of the parameter values considered in Fig. 3 is the system ultrasensitive.

**Fig 4:**
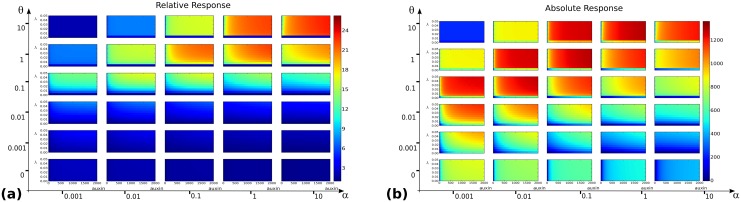
Zoom near *λ* = 0 of output landscapes as functions of auxin level *x* (abscissae) and balance between the two core mechanisms, parametrized by *λ* (ordinates). **(a)** Relative response *ρ*_*rel*_(*x*,*λ*). **(b)** Absolute response *ρ*_*abs*_(*x*,*λ*).

**Fig 5:**
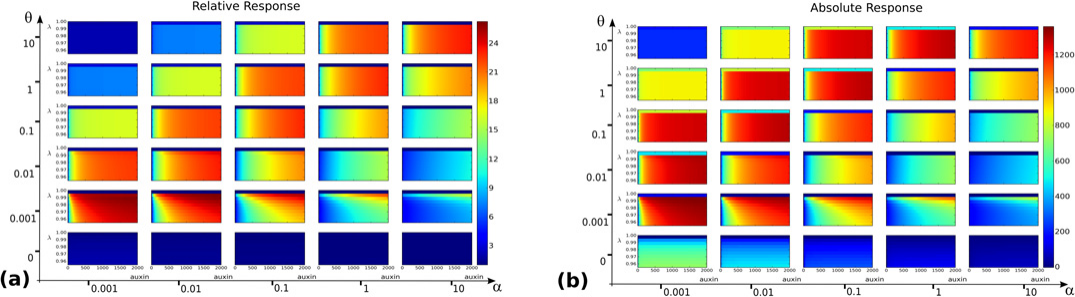
Zoom near *λ* = 1 of output landscapes as functions of auxin level *x* (abscissae) and balance between the two core mechanisms, parametrized by *λ* (ordinates). **(a)** Relative response *ρ*_*rel*_(*x*,*λ*). **(b)** Absolute response *ρ*_*abs*_(*x*,*λ*).

The previous observation suggests a closer inspection near the limit values *λ* ∈ {0,1}. It appears that these limits systematically correspond to a drastic decrease in the output functions, as shown in Figs. 4 and 5. On these figures on can see sharp transitions to lower values for the three functions *ρ*_*abs*_, *ρ*_*rel*_ and *σ* near *λ* = 0 and *λ* = 1. If the sharpness might look like the result of numerical inaccuracies, further increases of the resolution can show that they in fact correspond to a smooth, yet abrupt, decrease (data not shown). Here again, these features were found also in the cases A-H shown in S1 Additional Information.

Consequently, one global conclusion is that *λ* does not seem to be a crucial parameter for the system, insofar as it does not take one of the extreme values 0 or 1 which both strongly reduce all the considered output functions. In other words, the core mechanism underlying auxin signalling seems to be a combination were both sequestration and active repression are required for the response to be high and robust.

This led us to remove the *λ* parameter from later investigations, where we explore more finely the influence of other parameters on the different output functions, as detailed in the following. The results above, will be used to select values of *α* and *θ* rates in the following, with the aim to guarantee a fairly high level for all the output functions (provided *x* is not too low).

Because ARF:IAA dimers have already been considered, as they constitute the core of what we have termed sequestration, we now turn specifically to the influence of homodimers (in the sense that they are composed of proteins of the same family, IAA or ARF).

#### Role of ARF:ARF dimers

As ARF:ARF dimers have been found on promoters [23], a potential role of these dimers one may expect on intuitive grounds is to increase the steepness, or in fact the sensitivity of the transcriptional response.

In the response landscapes shown in Fig. 3 the sensitivity output is never greater than 1, i.e. the system is not ultrasensitive for the parameters explored in these landscapes in apparent contradiction with results suggesting that systems where a molecule (here IAA) sequestrates an other (here ARF) are known to present ultrasensitivity [32, 33].

Probably the strongest restriction imposed on the parameters used in Fig. 3 is the fact that all association (resp. dissociation) reactions have the same kinetic constant *α* (resp. *θ*). Although not a requirement, it has been observed in [32] that a disparity in decay rates can strengthen ultrasensitivity. This led us to consider alternative parameter values, where different binding reactions have distinct rates. None of our attempts showed ultrasensitive behaviour. Although the immense range of possible choices prohibited an exhaustive exploration, this indicates at least that ultrasensitivity may not be a dominant behaviour.

Finally, we investigated an intriguing possibility, suggested by recent experimental results on the structure of the domain III-IV of ARF and IAA that mediate protein-protein interactions. Several recent papers show indeed that ARF protein have the ability to regulate transcription with cooperativity, and can agglomerate as multimers of higher order than 2 proteins [4, 26, 27]. Even though the function of these structural properties would be a modelling research topic in itself, we considered a very simplified representation of these properties in our model. Namely, we introduced an additional parameter *n*_*A*_ representing the number of ARF proteins agglomerated in a multimer. This parameter can also be interpreted as a measure of cooperativity. The following alterations of Equation (5) were performed:

- terms *α*_*AA*_ *A* ^2^−*θ*_*AA*_ *D*_*AA*_ were replaced by $\alpha_{AA}A^{n_{A}}-\theta_{AA}D_{AA}$ in the equations $\dot{A}$ and ${\dot{D}}_{AA}$.
- terms *α*_*AG*_*A*__ *AG*_*A*_−*θ*_*AG*_*A*__ *G*_*AA*_ were replaced by *α*_*AG*_*A*__ *A* ^*n*_*A*_−1^ *G*_*A*_−*θ*_*AG*_*A*__ *G*_*AA*_ in the equations ${\dot{G}}_{A}$ and ${\dot{G}}_{AA}$.

These alterations are clearly phenomenological and a more realistic account would require the introduction of new variables for each multimer involving *p* ≤ *n*_*A*_ ARF proteins, see [34] for a related discussion. In this new formulation, *D*_*AA*_ does not represent ARF:ARF dimers, but multimers composed of *n*_*A*_ ARF proteins.

**Fig 6:**
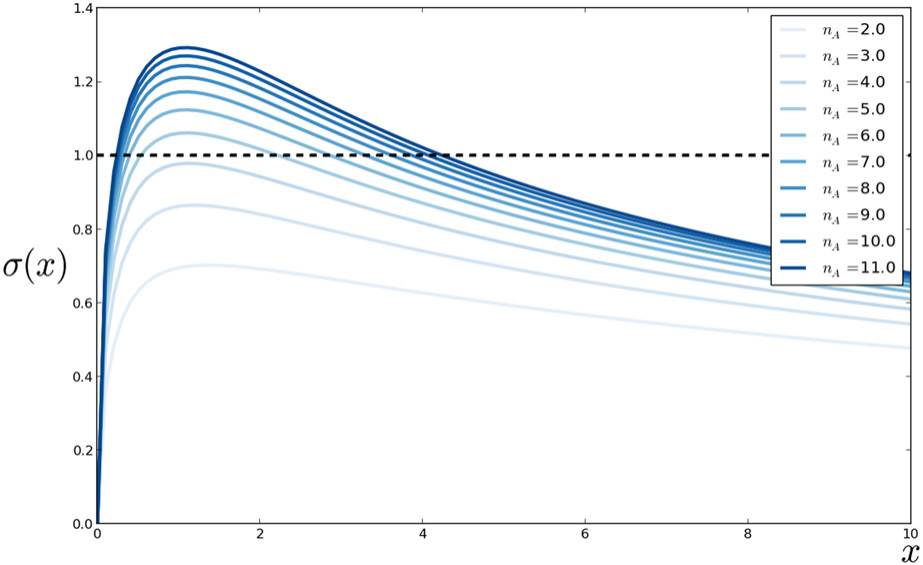
Sensitivity curve *σ*(*x*) as a function of the auxin level *x*, for *x* taking a sample of 100 values in the range [0, 10]. The association and dissociation parameters (*α*,*θ*) = (10,0.001) and the number of ARFs possibly bound in a polymer, denoted *n*_*A*_, increasing from 2 to 11 by unit increments.

As parameters like *n*_*A*_ are known to increase the steepness of the transcriptional response as a function of ARF, one may expect ultrasensitivity to occur for the full pathway for high values of *n*_*A*_. To assess the validity of this claim, we calculated the sensitivity *σ*(*x*) for a sample of values *x* ∈ [0, 300], for different choices of *α* and *θ* (common for all binding reactions). This led us to empirically select the values (*α*,*θ*) = (10,0.001), for which the sensitivity was higher. For this choice (corresponding to very strong dimer associations, i.e. a very low *K*_*d*_ = 10^−4^), we computed the curve (*x*,*σ*(*x*)) for increasing values of *n*_*A*_ as shown in Fig. 6. The sensitivity did raise above 1 for higher values of *n*_*A*_ and at lower values of *x*, below 10.

#### Role of IAA:IAA dimers

The role of IAA:IAA dimers is not intuitively obvious. This led us to consider a highly simplified model in which a single population of proteins is produced, degraded and can form homodimers, in order to derive first predictions which we then test in our complete model of the auxin pathway. Note also that the existence of higher orders IAA complexes [26, 27] has been suggested but has not been considered here for the sake of simplicity (but see discussion).

As is very often considered, we assume that dimers reach equilibrium at a faster rate than the production (transcription + translation) or degradation of proteins. This leads to the following equation (cf. Suppl. Info. for details):

$$\begin{array}{c} \frac{dI}{dt}=\delta \left(\pi_{I}-I\right)-\gamma I^{2}, \end{array}$$

where the parameter *γ* is proportional to the dimer association rate and *π*_*I*_ is the steady state value in absence of homodimerization. A first calculation shows that the unique steady state I* of this equation is a decreasing function of *γ*, and in particular it is always lower than *π*_*I*_ for *γ* > 0.

Since increasing *γ* results in a lower steady state value, a given percentage of this steady state might be attained quicker even though the system evolves at the same speed. In order to assess the response time we rescaled the system to ensure the steady state is 1 regardless of *γ*:

$$\begin{array}{c} \frac{dI}{dt}=\gamma +\delta -\delta I-\gamma I^{2}. \end{array}$$

Due to its simplicity, a closed form solution can be found for this equation, allowing to calculate a characteristic response time. From these calculations (cf. Suppl. Info.), one deduces that the characteristic time is a decreasing function of *γ*, which tends to 0 as *γ* → ∞. In other words, the stronger the homodimer formation rate, the faster the system reaches equilibrium (for instance after a perturbation), in principle arbitrarily fast if the dimers are strong enough.

The discussion above concerns a highly simplified description of the whole system, and is discussed only for it suggests a potential role of the IAA:IAA dimers improving the response time of the system. To confirm this suggestion, we return to the full model (5) and fixing all other parameters, assess the influence of *α*_*II*_ on the system’s response time.

More precisely, we varied all association and dissociation constants in the same coarse grid as in Fig. 3. For each of these values, we ran simulations of the model (5) with a constant input of auxin $\bar{x}=3000$ for a sample of values of the *α*_*II*_ association constant in the range [0, 100], recording the response times *τ*(*x*).

Since the analysis of the simplified model above considered a response time with a normalized steady state, we also calculated a *response speed*, defined as the ratio of the absolute response *ρ*_*abs*_ over the response time. When expressed as a ratio, this speed would for instance be reduced if the steady state is approached within the same time span, but has a lower value.

**Fig 7:**
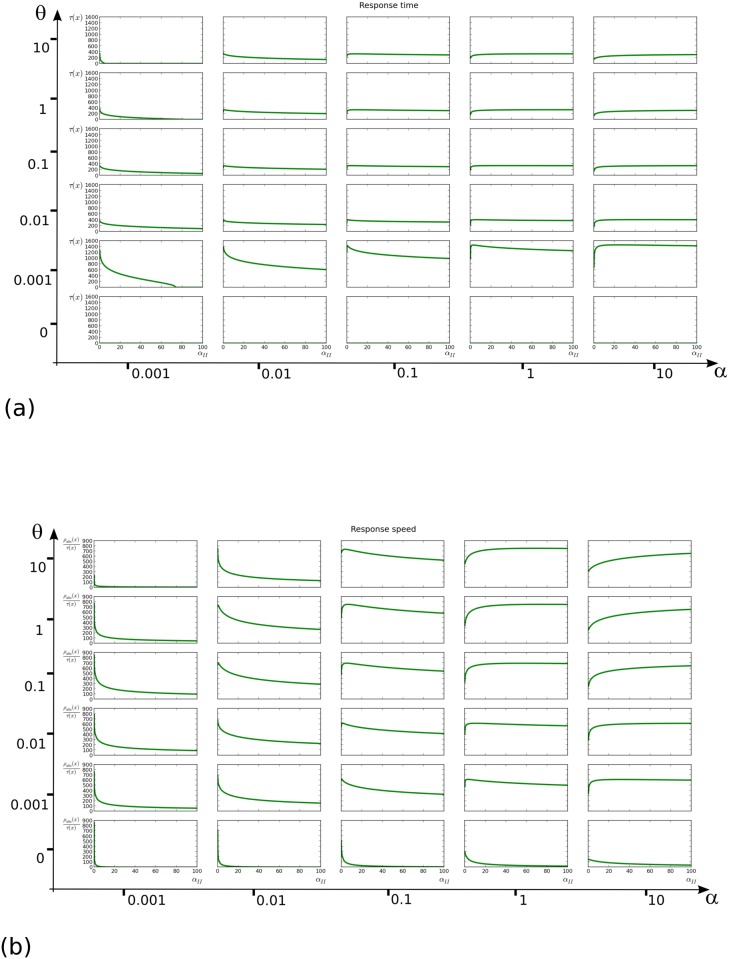
Time and speed of response as functions of *α*_*II*_. The grids correspond to distinct values of *α* and *θ*, which are the common association and dissociation constants of all binding processes in Equation (5), with the exception of *α*_*II*_ which is varied in each plot. **(a)** Response time vs. *α*_*II*_ **(b)** Response speed vs. *α*_*II*_. See main text for precise definitions of the response time and speed.

As anticipated, the response time did actually often increase with *α*_*II*_, see Fig. 7(a), but this was generally correlated with an increase of the steady state value (not shown). The response speed, on the other hand, displayed a clearer pattern shown in Fig. 7(b): for most (*α*,*θ*) in the region *θ* > 0 and *α* > 0.1 the system responded in shorter time (at higher speed) as *α*_*II*_ was increased and overall it appeared that any response with *α*_*II*_ > 0 was faster than with *α*_*II*_ = 0. This confirmed that the observation made on the simplified model above is still valid for wide ranges of parameters in our complete model.

This section suggests that the overall auxin signalling dynamics is faster when IAAs are able to form dimers, with the caveat that this requires other parameters to belong to specific (wide) ranges. This type of speed up induced by a nonlinear feedback term has been previously observed [35], although in that case it was self-activation, whereas we deal here with a process whose effect is ultimately self-inhibitory.

#### Forms of auxin induced degradation

As discussed in the first section it is assumed in our main model that dimers comprising an IAA unit can lose this unit upon auxin binding, releasing the other protein forming the dimer. However, other scenarii are also plausible. In particular, we have investigated the following alternative mechanisms:

- *(m1)* Dimers are not affected by auxin.
- *(m2)* Dimers bearing an IAA are entirely degraded by the proteasome as a consequence of the IAA binding TIR1 through auxin.

The mechanism *(m1)* may correspond to a situation where the structural conformation of IAA:X dimers prevents binding of auxin to the domain II of the IAA, whereas *(m2)* would account for ARF:IAA and IAA:IAA being obligate dimers.

In terms of the ODE model (5), *(m1)* can be modelled by suppressing all the terms *δ*_*II*_ *xD*_*II*_ and *δ*_*AI*_ *xD*_*AI*_ from the system. Note that in an extreme variant of this mechanism, dimers are not degraded at all (or at rate negligible compared to dissociation), in which case *δ*_*II*_ and *δ*_*AI*_ are simply set to zero.

For the mechanism *(m2)* on the other hand, the terms *δ*_*II*_ *xD*_*II*_ and *δ*_*AI*_ *xD*_*AI*_ are suppressed from the rates of *I* and *A*, but remain as decay terms in the rates of *D*_*II*_ and *D*_*AI*_.

To begin exploring the properties of these two mechanisms, we computed the same landscapes as in Fig. 4 (see S2 Additional Information) for the two alternative models *(m1)* (for its extreme variant) and *(m2)*. The overall effect for *(m1)* was not obvious, all the main characteristics discussed in the previous section remaining unchanged.

On the other hand, in the case *(m2)* a noticeable effect could be observed: in some parameter ranges, auxin could act as repressor, despite our default situation involving an activator ARF. These exploratory simulations seemed to indicate that the phenomenon was mostly seen near *λ* = 1, i.e. in a regime where the effect of auxin is mostly due to ARF being sequestered in ARF:IAA dimers. In agreement with the previous section, we sought to reproduce this phenomenon in a model without *λ* (i.e. with both core mechanisms).

**Fig 8:**
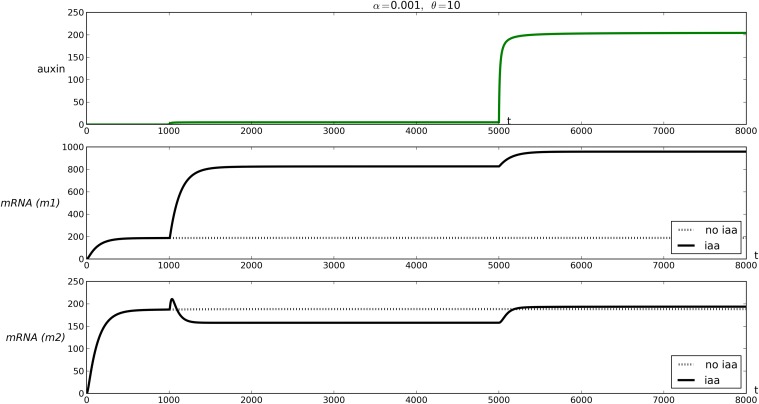
Time courses of auxin, and mRNA for two modes of auxin induced IAA degradation. First row: auxin concentration. Second and third rows: mRNA concentration (variable *R*) for models *(m1)* and *(m2)* respectively, in full line. The dotted lines represent the evolution of mRNA if the input of auxin is not included (i.e. *x* = 0 for all *t* ≥ 0). Two inputs of auxin are performed at time *t* = 1000 and *t* = 5000, the second of higher amplitude. *α* = 0.01, *θ* = 10.0 and all other parameters are as in Table 1 except *h* = 0 and *h*_*AI*_ = 1.

A regime that would be comparable to high *λ* values is one in which the repressive action of ARF:IAA dimer is weak, which corresponds to *h* being close to or maybe higher than *h*_*AI*_. We thus considered a variant of the default parameters shown in Table 1, fixing *h* = 0 and *h*_*AI*_ = 1 (reversing the default values) and leaving all other parameters unchanged. For this choice we found, as in the landscapes discussed above, that auxin could act as a repressor in the case of model *(m2)*, but not in model *(m1)*. Some typical curves are shown in Fig. 8. Similar curves were found for pairs (*α*,*θ*) such that the dissociation constant $K_{d}=\frac{\theta }{\alpha }\geq 1000$. For other values of the dissociation constants, the absolute response was systematically higher with mechanism *(m1)* than with *(m2)*, suggesting that even in a regime where auxin activates the transcription of its targets, its efficacy is dampened in the case of *(m2)*. Remarkably, the repressor effect was only observed for a limited range of auxin levels and in every model transcription is increased upon high inputs of auxin.

An intuitive explanation of the repressive action of auxin with degradation mechanism *(m2)* is that ARF:IAA degradation (induced by auxin) may result in an overall decrease of the ARF population. This can result in a reduction of the transcriptional activation by ARFs and their dimers. If the basal level of transcription is itself low, the end result of an input of auxin might be a repression of the target gene, even if the main population of ARF is composed of activators. Note that this phenomenon does not appear with other forms of dimer degradation and is furthermore limited to a peculiar set of parameter values (high *K*_*d*_, i.e. weak dimers).

#### Role of feedback

Our primary definition of feedback is due to IAA proteins having genes including ARF binding sites. Beyond this primary loop, there exists experimental evidence that some ARF proteins are also regulated by auxin [17]. In the case of activator ARFs, this results in a positive feedback loop, whereby an increase of ARF level ultimately increases further the production of ARF proteins (by inducing an increase on the transcriptional activation of auxin responsive genes, which include ARF coding genes).

Feedback loops have a crucial influence on the qualitative dynamics of a system, and it is established that the presence of a negative feedback loop (resp. positive feedback) is necessary for the occurrence of periodic solutions, i.e. oscillations (multiple steady states, i.e. multistability) [36–38]. These results are necessary conditions only, and the presence of bistability or oscillations, though it can be excluded in absence of any feedback loop, can only be confirmed by an analysis which is often non trivial.

In fact, the so-called mixed feedback loop is essentially a sequestration system with an additional feedback loop, and has been shown in [39] to have the potential for bistability when the loop is positive and oscillatory behaviour when the loop is negative. Although suggestive, this result can only be transposed to our situation with great care, as the auxin pathway presents several differences with the mixed feedback loop. Firstly, in the latter it is assumed that only the (sequestered) monomer is a regulator, whereas both ARF and ARF:IAA regulate transcription. Secondly, in the auxin pathway dimers are formed not only between ARF and IAA but also amongst each of these two families.

Concerning the negative feedback, a previous study has shown that the auxin pathway could in principle present stable oscillations [40]. Since this study involved a model that had a similar level of detail as our present system, we did not attempt a thorough exploration of oscillations, as it would have been redundant with [40].

We considered the role of a positive feedback on ARF+, which we implemented as follows in Equations (5):

- *π*_*A*_ is replaced by *π*_*A*_ *R*, becoming dependent on auxin regulated transcription,
- the production rate of *I* is a constant *π*_*I*_.

**Fig 9:**
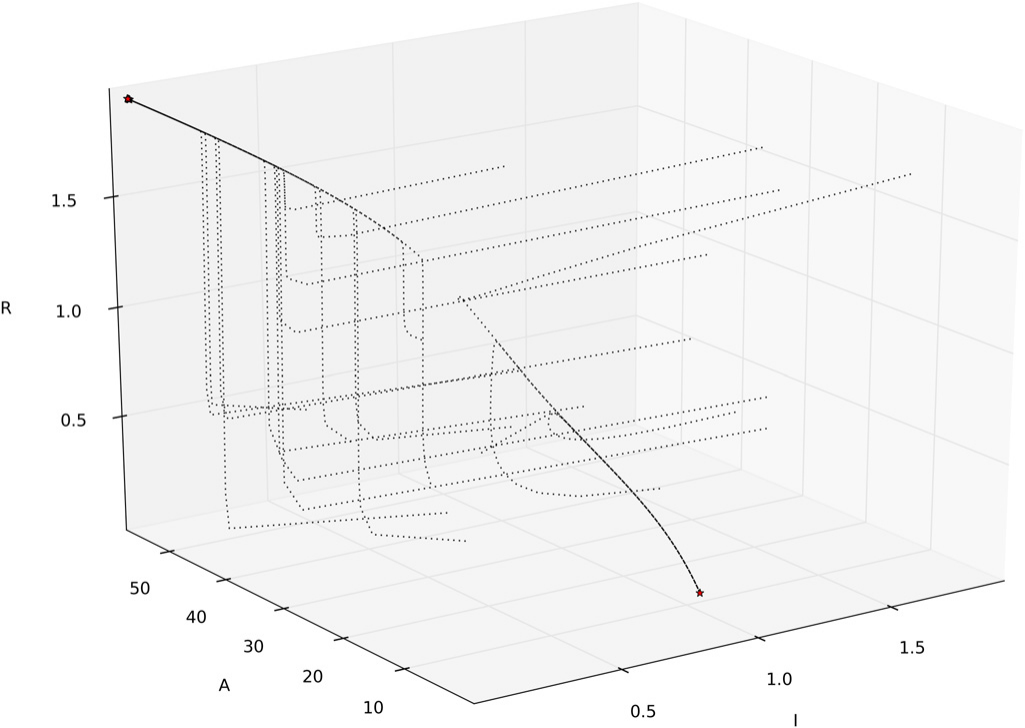
Illustration of the bistable behaviour with positive feedback (via ARF) in model (5)–(7) (in S1 Text). Trajectories are shown for 20 randomly chosen initial conditions. The two steady states are marked with stars ⋆. Parameter used: $\left[\gamma_{II},\gamma_{IA}^{\pm },\delta_{A},\delta_{I},\pi_{I},\pi_{A},\omega_{0},\omega_{1},\omega_{2}]=[1,1,0.003,0.05,0.8,0.5,2,1,1\right]$.

The last assumption was not strictly speaking required to investigate positive feedback, but was used as a simplifying assumption which allowed to perform analytical calculations. Namely, in a specific parameter regime where all dimers are supposed to occur more rapidly than protein production and degradation (using a quasi-steady state assumption), we were actually able to characterize analytically some parameter values for which the system could admit multiple equilibria, see S1 Text and Fig. 9. These results were obtained under a quasi-steady state assumption, only valid in fairly specific regions of the parameter space of (5).

## Discussion

Through a modelling approach, we have been able to analyze the contribution to the response dynamics of most of the topological features of the ARF/IAA signalling pathway controlling transcription in response the plant hormone auxin, thus providing key insights into the regulatory potential of this signalling pathway crucial to plant development.

First, our results predict that sequestration of ARF by IAA away from promoters and active repression through recruitment of TPL/TPR by IAA on ARF-bound promoters are both required for a full relative and absolute response of the system. This thus suggests that the IAAs have both a passive and an active role that act synergistically in the repression of auxin-induced genes. As dimerization and recruitment of TPL/TPR are linked to different and largely independent conserved domains of IAA proteins (respectively domain III-IV and domain I), this prediction could be tested by comparing the repressive capacity of partially truncated proteins. A contribution of sequestration of ARFs by IAAs also opened the possibility that this mechanism could lead to hypersensitivity of the auxin signalling pathway [32, 33], a property that could generate bistability in the presence of a positive feedback loop. However, we could not observe any hypersensitivity linked to sequestration in our model. One important difference between previous analyses pointing at a role for sequestration in hypersensitivity [32, 33] and the present paper is the choice of input and output variables. We considered auxin as an input and the concentration of transcribed mRNA as output. In the cited studies, the output is the concentration of the sequestered molecule in its free form (here, ARF), while the input is the total concentration of this molecule, including its sequestered form (here, ARF + ARF:IAA). In a way, this I/O interpretation could be seen as an intermediate module in our system: auxin can be seen as acting upstream of IAA proteins (and thus the total population ARF + ARF:IAA), while mRNA transcription takes place downstream of ARF.

In general, the sensitivity of a linear chain of dependency between variables, say *x* → *y* → *z*, is the product of sensitivities of the subchains *x* → *y* and *y* → *z* (as results from the definition of sensitivity and the rules of calculus). With these notations, it follows that for *z* to be ultrasensitive as a function of *x* it is necessary, but not sufficient, that one of the two chains at least be ultrasensitive (*σ*_*zx*_ = *σ*_*zy*_ *σ*_*yx*_ cannot be > 1 if both *σ*_*zy*_ ≤ 1 and *σ*_*yx*_ ≤ 1, but one may have *σ*_*yx*_ > 1 and *σ*_*zx*_ ≤ 1). Hence, our observations are not in contradiction with the previous literature. The fact that we consider a longer chain of reactions between the input and the output is not the only explanation of why we did not observe ultrasensitivity. Importantly, our system also presents feedback, which significantly adds to the complexity of the analysis. In particular, and as pointed out before, a system called the *mixed-feedback loop* is essentially a sequestration system as studied in [32, 33] with the addition of a feedback loop. This has been shown to present a rich variety of behaviours [39], and the extent to which it affects sensitivity could be the subject of future work.

It is also worth noticing here that our model represents the perception of auxin by TIR1 receptors in a simplified manner, and this module has been shown experimentally to affect sensitivity in the auxin pathway [41].

The role for dimerization and multimerization in transduction pathways remains largely unknown in vivo so far. Our work leads to important predictions on the contribution of protein-protein interactions that highlight their fundamental role in governing auxin-induced transcriptional dynamics and that could thus be generally relevant for other transduction pathways. We show that IAA homodimerization has the potential to modulate the response time of the transduction and thus share known properties with negative feedback loops [42]. Notably, our results indicate that the absence of IAA-IAA interactions would lead to a severe decrease in the speed of the induction of transcription, thus identifying a clear function for this topological element of the pathway. We suggest that this effect is due to homodimerization acting as a buffer on IAA concentration. Similarly to sequestration, we could not find any evidence of hypersensitivity linked to ARF dimerization on promoters despite a potential cooperative effect. However, we provide evidence that the capacity of ARFs to generate higher order polymers in solution [26, 27] could be important for the generation of an hypersensitive response by increasing the steepness of the expression of auxin target genes. A full exploration of the role of ARF multimers would require a much more thorough analysis but our work serves as a proof of principle of the importance of these polymers. Polymers for IAAs have also been found in solution. Such multimers have not yet been reported in vivo (nor for IAAs neither for ARFs) but it would be interesting to use theoretical approaches such as ours to explore whether IAA polymers can also potentialize further the capacities of IAAs to speed up the response to auxin.

Finally, we also found that by degrading ARF-IAA dimers, the transduction pathway has the potential to turn the system into a repressive regime, even if the ARF population interacting with IAA is composed mostly of activators. This repression was only observed for parameter values corresponding to weakly bound dimers. Given the diversity of ARFs and IAAs, such parameter values may be representative of existing ARF and IAA proteins, indicating another potential repressive mode of auxin action on its targets that has not been identified so far biologically.

All our attempts at finding bistability were only successful in the peculiar parameter regimes described in the S1 Text. Even though this partial exploration cannot be invoked to definitely rule out bistability in other regimes, it might be that positive feedback alone is not an efficient way to generate bistability in this system. Here again, the control of sensitivity by ARF multimerization could be a mechanism which, combined with a feedback loop on ARF, leads to bistable dynamics.

In [17], the authors explored the function of the ARF5 positive feedback loop present in an instance of the auxin pathway that is primarily controlled by ARF5 and IAA12. While the authors proposed that this feedback loop could lead to a switch, this system presented bistability only in some restricted parameter regimes. As the authors used a phenomenological ODE model, the bistability they observed could be linked to intrinsic properties of their model explaining the discrepancies with a model including molecular details such as the one used in this work. From our result and the result of [17] one may hypothesize that a higher sensitivity, entailed by ARF multimerization, in conjunction with a feedback loop would allow for bistability to occur robustly. This would have to be confirmed through modelling, but more importantly by experimental means.

**Fig 10:**
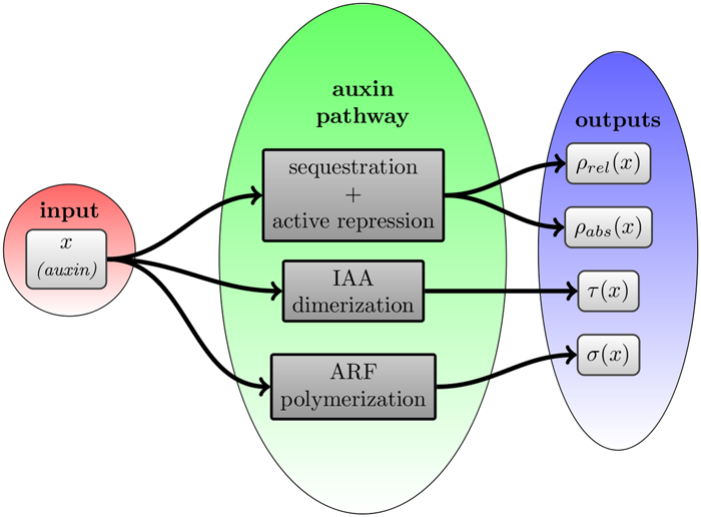
Module specific regulation. Different modules regulate specific outputs of the pathway.

In conclusion, our analysis allows in a way to partially “untangle the wires” of the auxin pathway, as schematized in Fig. 10. Although the number of parameters of the model excluded their complete exploration, and thus a rigorous and systematic test of the robustness of each of these behaviours, it seems that the contribution of all parameters can be robustly combined to modulate every aspect of the perception of auxin, at the cellular level.

This result is also valuable in general for signal transduction pathways and in particular for plant hormone gibberellin and jasmonate pathways (which are crucial for the regulation of growth and development in response to the environment) that share some topological features with the auxin pathway [43]. It is possible that such topology is the product of natural selection, in that each of the topological features we studied seems to have a precise function in hormone perception. Importantly, this theoretical work leads to predictions that can be experimentally tested as pointed out already before. For this purpose, synthetic biology approaches using reconstruction of the auxin signalling pathway in yeast are a powerful way to de-contextualize the pathway from plant development and study its autonomous dynamics [44, 45]. Such a system could be used to test our predictions before further analysis *in planta*. We have already discussed ways to explore the importance of sequestration. The diversity of IAA and ARF also further gives the possibility to explore the effect of changing ARF:IAA and IAA:IAA affinities (and thus the interaction properties at the heart of this pathway), although biochemical analyses would be needed to measure the affinity constant of at least a few proteins. Interestingly, the recent structural data on ARFs [4, 26, 27] provide a framework for modifying the interaction properties between IAAs and ARFs and to modify different topological features of the signalling pathway to analyse their contribution in the artificial situation of the yeast but also in plants.

Finally, our work suggest that the activity of the auxin signalling pathway could be modulated by a large variety of mechanisms targeting the different topological features we have studied. Such regulation could be crucial during development or in response to the environment. This is highlighted by the recent demonstration that post-transcriptional modification of ARF7 and ARF19 modifying their interaction capacities with IAAs provide an important level of regulation during auxin-induced lateral root development [46].

## Methods

Instead of defining each individual variable, we adopt the following nomenclature:

- *I* and *A* respectively denote the concentration of IAA and ARF proteins.
- *D*_*XY*_ generically denote the concentration of an X:Y dimer.
- *R* denotes the concentration of IAA mRNA.
- *G*, *G*_*A*_, *G*_*AA*_ and *G*_*AI*_ respectively denote the probability of the promoter being free, bound to an ARF protein or bound to an ARF:ARF or ARF:IAA dimer.
- We use *x* to denote the concentration of auxin. Note that because of our simplifying assumptions regarding auxin perception, *x* is more accurately interpreted as a combined rate including the level of auxin and its receptors, but will often be referred to as “auxin level” in the following for simplicity.

Then, we use mass action law for all the binding reactions mentioned above. We denote association (resp. dissociation) rates by parameters of the form *α*_*XY*_ (resp. *θ*_*XY*_), where *X* and *Y* denote the two subunits of a molecule whose physical interaction creates a new complex.

The remaining parameters include degradation rates, denoted *δ*_*I*_, *δ*_*A*_, *δ*_*AA*_, *δ*_*AI*_ and *δ*_*II*_ for IAA, ARF, ARF:ARF, ARF:IAA and IAA:IAA respectively. The production rates of IAA and ARF are *π*_*I*_ and *π*_*A*_ respectively.

Finally, the transcription rates of the promoter in its different configurations are denoted *h*, *h*_*A*_, *h*_*AA*_ and *h*_*AI*_ for ARF, ARF:ARF and ARF:IAA respectively. Whether the considered is an activator or repressor ARF can be represented using respectively high or low values, relative to *h*, for both the transcription rates *h*_*A*_ and *h*_*AA*_. Our default model considers an activator ARF, i.e. high values of both these transcription rates, but variations will be considered in the following.

Note that as a general rule, all parameters will be assumed to be positive, unless the case of a particular parameter approaching zero is explicitly considered. The actual values of the different parameters is discussed below.

The main hypotheses listed in the first section lead to the following system of ODE:

$$\begin{array}{c} \left\{\begin{array}{ccc} \frac{dI}{dt} & = & \;\pi_{I}R+2\theta_{II}D_{II}-2\alpha_{II}I^{2}+\theta_{AI}D_{AI}-\alpha_{AI}IA+\;\theta_{G_{A}I}G_{AI}-\alpha_{G_{A}I}IG_{A}\\ & & +\delta_{II}D_{II}\kappa_{x}x-\delta_{I}\left(1+\kappa_{x}x\right)I\\ \frac{dA}{dt} & = & \pi_{A}+2\theta_{AA}D_{AA}-2\alpha_{AA}A^{2}+\theta_{AI}D_{AI}-\alpha_{AI}IA+\theta_{AG}G_{A}-\alpha_{AG}AG\\ & & +\;\theta_{AG_{A}}G_{AA}-\alpha_{AG_{A}}AG_{A}+\delta_{AI}D_{AI}\kappa_{x}x-\delta_{A}A\\ \frac{dD_{II}}{dt} & = & \alpha_{II}I^{2}-\theta_{II}D_{II}-\delta_{II}\left(1+\kappa_{x}x\right)D_{II}\\ \frac{dD_{AI}}{dt} & = & \alpha_{AI}IA-\theta_{AI}D_{AI}+\theta_{D_{AI}G}G_{IA}-\alpha_{D_{AI}G}GD_{AI}-\delta_{AI}\left(1+\kappa_{x}x\right)D_{AI}\\ \frac{dD_{AA}}{dt}\; & = & \alpha_{AA}A^{2}-\theta_{AA}D_{AA}+\theta_{D_{AA}G}G_{AA}-\alpha_{D_{AA}G}GD_{AA}-\delta_{AA}D_{AA}\\ \frac{dG}{dt} & = & \theta_{AG}G_{A}-\alpha_{AG}AG+\theta_{D_{AI}G}G_{AI}-\alpha_{D_{AI}G}GD_{AI}+\theta_{D_{AA}G}G_{AA}-\alpha_{D_{AA}G}GD_{AA}\\ \frac{dG_{A}}{dt} & = & \alpha_{AG}AG-\theta_{AG}G_{A}+\theta_{G_{A}I}G_{AI}-\alpha_{G_{A}I}IG_{A}+\theta_{AG_{A}}G_{AA}-\alpha_{AG_{A}}AG_{A}\\ \frac{dG_{AA}}{dt} & = & \alpha_{AG_{A}}AG_{A}-\theta_{AG_{A}}G_{AA}+\alpha_{D_{AA}G}GD_{AA}-\theta_{D_{AA}G}G_{AA}\\ \frac{dG_{AI}}{dt} & = & \alpha_{G_{A}I}IG_{A}-\theta_{G_{A}I}G_{AI}+\alpha_{D_{AI}G}GD_{AI}-\theta_{D_{AI}G}G_{AI}\\ \frac{dR}{dt} & = & hG+h_{A}G_{A}+h_{AA}G_{AA}+h_{AI}G_{AI}-\delta_{R}R \end{array}\right. \end{array}$$ (5)

Note that the sum *G*+*G*_*A*_+*G*_*AA*_+*G*_*AI*_ has zero derivative and one of these equations is thus redundant. As mentioned above, we interpret this quantities as probabilities of the promoter being in its different states, and thus consider that *G*+*G*_*A*_+*G*_*AA*_+*G*_*AI*_ = 1 hereafter.

We can also represent the system graphically. Using the following convention for reversible or irreversible reactions $A+B\overset{\theta }{\underset{\alpha }{⇌}}A:B$ and $A+B\overset{k}{\to }A:B$ respectively: 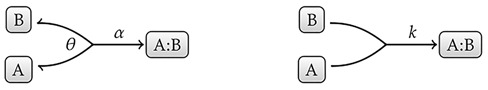


Then, representing each reactant only once and using the hypotheses described above, the reactions that can take place in the auxin signalling pathway can be depicted by the graph in Fig. 1.

As parameters are varied in different ranges through the paper, a default set of values is fixed and serves as a reference for all values not specified explicitly in the main paper. These values are chosen according to published experimental data when available, or to physically or chemically plausible ranges when no experimental data was found in the literature. Based on the known values detailed below, the following physiologically relevant units have been chosen: time is expressed in minutes and concentrations are expressed in nM. Being interpreted as probabilities, the variables *G*, *G*_*A*_, *G*_*AA*_ and *G*_*AI*_ are dimensionless. It follows that all protein and mRNA decay rates are expressed in nM.min^−1^, protein-protein association (resp. dissociation) rates are in nM^−1^.min^−1^ (resp. min^−1^), protein-promoter association (resp. dissociation) rates are in min^−1^ (resp. nM.min^−1^), and finally the production rates *π*_*I*_ and *π*_*A*_ are respectively in min^−1^ and in nM.min^−1^.

The half-life of various IAA proteins have been measured experimentally under different levels of auxin [30]. The measured values range between less than 15 minutes and more than 20h, although most of them were shorter than 1h. These half-life values were approximately halved upon auxin induction. Also ARF1 half-life has been measured [47] and is of 3-4h, which we chose as reference value for all ARF proteins. As dimers are known to be often more stable than the monomers they are composed of [48], we made the assumption that dimers were degraded at a rate similar to the more stable of monomers, i.e. ARFs, and thus share the same degradation rate.

Also, [49] provides mRNA half-life of essentially all genes of *Arabidopsis thaliana*, from which we extracted the values of the IAA coding genes. Their average half-life was of ≈ 4h30, which we took as a reference to fix the *δ*_*R*_ value.

Finally, none of the association or dissociation constants are known experimentally for the auxin pathway. For this reason, the corresponding parameters have been varied over several orders of magnitude, within ranges known to be meaningful in other experimental systems [32, 33].

Expressed in the units described earlier, the available data just discussed led us to the reference set of parameters shown in Table 1.

## Supporting Information

S1 Text Contains details of calculations mentioned in the main text. (PDF)

S1 Additional Information Contains response landscapes corresponding to the parameter exploration discussed in Section **Influence of parameters on I/O behaviour**. (PDF)

S2 Additional Information Contains response landscapes for models M1 and M2 discussed in Section **Forms of auxin induced degradation**. (PDF)
