# Correction: A Modular Analysis of the Auxin Signalling Network

**DOI:** 10.1371/journal.pone.0131622

**Published:** 2015-06-24

**Authors:** Etienne Farcot, Cyril Lavedrine, Teva Vernoux

Fig 3 is incorrect. Please see the corrected Fig 3 here.

**Fig 3:**
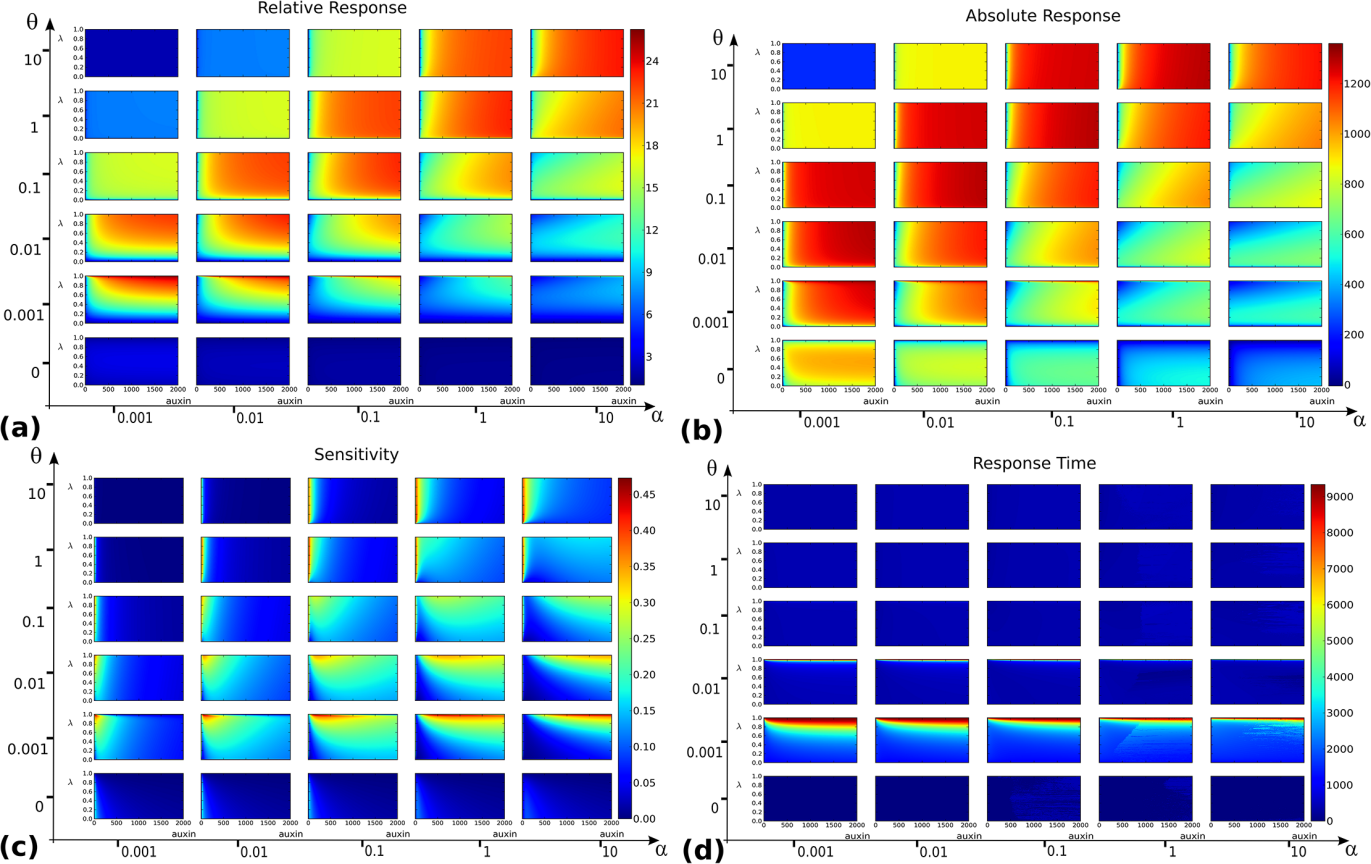
Output landscapes as functions of auxin level *x* (abscissae) and balance between the two core mechanisms, parametrized by *λ* (ordinates). **(a)** Relative response *ρ*_*rel*_(*x*,*λ*). **(b)** Absolute response *ρ*_*abs*_(*x*,*λ*). **(c)** Sensitivity *σ*(*x*,*λ*). **(d)** Response time *τ*(*x*,*λ*). For each of these functions, the grid of values (*α*,*θ*) Є {0.001,0.01,0.1,1,10}×{0,0.001,0.01,0.1, 1,10}is considered and for each landscape (*x*,*λ*) span a 200×200 regular grid on the rectangle [0, 2000]×[0, 1].
