# Supplementary material for: A Modular Analysis of the Auxin Signalling Network
Source: PLoS One. 2015 Mar 25;10(3):e0122231. doi: 10.1371/journal.pone.0122231 (PMC4373724; doi:10.1371/journal.pone.0122231)
Supplement: S1 Text — (PDF) [file pone.0122231.s001.pdf]

A modular analysis of the auxin signalling network Supplementary Information

E. Farcot, C. Lavedrine, T. Vernoux

Response speed-up by homodimerization

To get some analytical insight into the potential role of IAA:IAA dimer formation, we consider the sole IAA dynamics, and compare the cases when homodimers form or not. In equations, we consider the populations of monomers I and dimers D_{II}:

$$\frac{dI}{dt} = \pi_I \delta + \theta D_{II} - \alpha I^2 - \delta I, \qquad \frac{dD_{II}}{dt} = \alpha I^2 - \theta D_{II} - \delta_{II} D_{II}.$$

The choice of a production rate written as $\pi_I \delta$ is designed to ensure that the (unique) equilibrium found in absence of homodimerization is equal to the single parameter π_I.
Assuming for simplicity that the dimers are at equilibrium (as would occur for instance with large α, θ), we find:

$$D_{II} = \frac{\alpha}{\theta + \delta_{II}} I^2 \quad \text{and} \quad \theta D_{II} - \alpha I^2 = -\delta_{II} D_{II} = \frac{-\delta_{II}\alpha}{\theta + \delta_{II}} I^2.$$

Using these relations gives a unique simplified ODE:

$$\frac{dI}{dt} = \delta(\pi_I - I) - \gamma I^2, \qquad \text{where} \qquad \gamma = \frac{\delta_{II}\alpha}{\theta + \delta_{II}}.$$

Two steady states are found by solving a quadratic equation, one of which is positive:

$$I^* = \frac{-\delta + \sqrt{\delta^2 + 4\delta\gamma\pi_I}}{2\gamma}. \tag{1}$$

This steady state I^* is always smaller than the value π_I found for $\gamma = 0$:

$$\pi_I - I^* = \frac{2\pi_I\gamma + \delta - \sqrt{\delta^2 + 4\delta\gamma\pi_I}}{2\gamma} > \frac{2\pi_I\gamma + \delta - \sqrt{(\delta + 2\gamma\pi_I)^2}}{2\gamma} = 0.$$

Moreover, it can be shown by differentiating I^* that it decreases as a function of γ:

$$\frac{\partial I^*}{\partial \gamma} = \frac{\delta\left(\sqrt{\delta^2 + 4\gamma\delta\pi_I} - \delta - 2\gamma\pi_I\right)}{2\gamma^2\sqrt{\delta^2 + 4\gamma\delta\pi_I}} < \frac{\delta\left(\sqrt{(\delta + 2\gamma\pi_I)^2} - \delta - 2\gamma\pi_I\right)}{2\gamma^2\sqrt{\delta^2 + 4\gamma\delta\pi_I}} = 0.$$

So, the steady state value is maximal without homodimerization and decreases as the latter becomes more prominent.

As we proceed to assess the influence of γ on the response time of the system, we rescale the production rate of I to ensure that steady state value is always 1 regardless of γ, i.e. we seek $\widetilde{\pi}_I$ such that

$$\frac{-\delta + \sqrt{\delta^2 + 4\delta\gamma\widetilde{\pi}_I}}{2\gamma} = 1 \iff \widetilde{\pi}_I = 1 + \frac{\gamma}{\delta}.$$

Note that in the limit $\gamma \to 0$ this gives $\widetilde{\pi}_I = 1$ as expected. So, we are now considering the rescaled system

$$\frac{dI}{dt} = (\gamma + \delta) - \delta I - \gamma I^2. \tag{2}$$

Let us fix $I(0) = 0$ for simplicity in the following. For this initial condition, it is possible to derive a closed form solution for Equation (2)[1]:

$$I(t) = \frac{(\gamma+\delta)\left(1 - e^{-(\delta+2\gamma)t}\right)}{\gamma+\delta+\gamma e^{-(\delta+2\gamma)t}}. \tag{3}$$

Using this closed form solution, it is also possible to compute the time taken to reach a given percentage $0 < \rho < 1$ of the equilibrium, i.e. the non-negative time τ_ρ such that the solution $I(t)$ to the equation above with $I(0) = 0$ verifies:

$$I(\tau_\rho) = \rho.$$

Then, a direct calculation gives

$$\tau_\rho = \frac{1}{\delta+2\gamma}\log\left(1 + \frac{\rho(\delta+2\gamma)}{(1-\rho)(\gamma+\delta)}\right), \tag{4}$$

which takes the value $\frac{1}{\delta}\log\left(\frac{1}{1-\rho}\right)$ when $\gamma = 0$. One can show in fact that this limit is an upper bound and τ_ρ decreases with γ for any value of ρ (see below). From the expression above, one can also verify that $\lim_{\gamma\to\infty}\tau = 0$.

Proof that τ_ρ decreases as a function of γ.
We compute explicitly its partial derivative of τ_ρ, Equation (4), with respect to γ:

$$\frac{\partial\tau_\rho}{\partial\gamma} = \frac{2}{(2\gamma+\delta)^2}\log\left(1 - \frac{\rho(2\gamma+\delta)}{(1+\rho)\gamma+\delta}\right) + \frac{\rho\delta}{(2\gamma+\delta)(\gamma+\delta)\left((1+\rho)\gamma+\delta\right)}$$

which has the same sign as

$$\frac{(2\gamma+\delta)^2}{2}\frac{\partial\tau_\rho}{\partial\gamma} = \log\left(1 - \frac{\rho(2\gamma+\delta)}{(1+\rho)\gamma+\delta}\right) + \frac{\rho\delta(2\gamma+\delta)}{2(\gamma+\delta)\left((1+\rho)\gamma+\delta\right)}.$$

Then, from $\log(1-x) < -x$ for all $0 < x < 1$ we find

$$\begin{aligned}\frac{(2\gamma+\delta)^2}{2}\frac{\partial\tau_\rho}{\partial\gamma} &< -\frac{\rho(2\gamma+\delta)}{(1+\rho)\gamma+\delta} + \frac{\rho\delta(2\gamma+\delta)}{2(\gamma+\delta)\left((1+\rho)\gamma+\delta\right)} \\ &= \frac{\rho(2\gamma+\delta)}{(1+\rho)\gamma+\delta}\left(-1 + \frac{\delta}{2(\gamma+\delta)}\right) \\ &= \frac{\rho(2\gamma+\delta)}{(1+\rho)\gamma+\delta}\cdot\frac{-(2\gamma+\delta)}{\gamma+\delta}.\end{aligned}$$

And thus $\dfrac{\partial\tau_\rho}{\partial\gamma} < \dfrac{-2\rho}{\left((1+\rho)\gamma+\delta\right)(\gamma+\delta)} < 0.$ □

Positive feedback and bistability

As discussed in the main text we implemented feedback on ARF+ as follows

- the production rate if I is a constant π_I,
- π_A is replaced by $\pi_A R$.

[1] Calculated with the aid of sage, see `http://www.sagemath.org/`

Furthermore, we considered a specific parameter regime for the main ODE system, which made some analytic calculations feasible. Namely, we supposed the following:

- the formation of all dimers and promoter-protein complexes are supposed at steady state
- ARF:ARF dimers form at a negligible rate, i.e. $\alpha_{AA} \approx 0$ and $\alpha_{AG_A} \approx 0$.

Under these assumptions, only the three variables A, I and R are non-steady, and straightforward calculations show that their dynamics follow an ODE system of the from

$$\frac{dI}{dt} = \pi_I - \gamma^{+}_{AI}(x)IA - \gamma_{II}(x)I^2 - \delta_I(x)I \tag{5}$$

$$\frac{dA}{dt} = \pi_A R - \gamma^{-}_{AI}(x)IA - \delta_A A \tag{6}$$

$$\frac{dR}{dt} = \frac{\omega_0 A}{1 + \omega_1 A + \omega_2 AI} - \delta_R R, \tag{7}$$

where

$$\omega_0 = \frac{h_A \alpha_{AG}}{\theta_{AG}}, \qquad \omega_1 = \frac{\alpha_{AG}}{\theta_{AG}}, \qquad \omega_2 = \frac{\alpha_{AG}\alpha_{G_AI}}{\theta_{AG}\theta_{G_AI}},$$

and

$$\gamma_{II}(x) = \frac{-\delta_{II}(2 + \kappa_x x)\alpha_{II}}{\theta_{II} + \delta_{II}(1 + \kappa_x x)}$$

$$\gamma^{+}_{AI}(x) = \frac{-\delta_{AI}\alpha_{AI}}{\theta_{AI} + \delta_{AI}(1 + \kappa_x x)}$$

$$\gamma^{-}_{AI}(x) = \frac{-\delta_{AI}\alpha_{AI}(1 + \kappa_x x)}{\theta_{AI} + \delta_{AI}(1 + \kappa_x x)}.$$

To consider the possible occurrence of multiple stable equilibria, let us compute the steady state equations of this system. Firstly,

$$A = \frac{\pi_A R}{\delta_A + \gamma^{-}_{AI}(x)I}$$

and this can be injected in the last equation to give

$$\delta_R \pi_A(\omega_1 + \omega_2 I)R^2 + (\delta_A + \gamma^{-}_{AI}(x)I - \omega_0 \pi_A)R = 0,$$

in other words $R \in \left\{0, \frac{\omega_0\pi_A - \delta_A - \gamma^{-}_{AI}(x)I}{\delta_R\pi_A(\omega_1 + \omega_2 I)}\right\}$ are two steady state solutions for (7). If there exists a steady state solution I to (5) such that the nonzero steady state R is positive, the system (5)-(7) is bistable.

As performing these computations analytically is not feasible, we simulated numerically solutions of (5)-(7) for various parameters, using the formulas above as a guide for intuition. We found some cases where bistability was occurring, see Figure 10.
