# Supplementary material for: A Modular Analysis of the Auxin Signalling Network
Source: PLoS One. 2015 Mar 25;10(3):e0122231. doi: 10.1371/journal.pone.0122231 (PMC4373724; doi:10.1371/journal.pone.0122231)
Supplement: S1 Additional Information — (PDF) [file pone.0122231.s002.pdf]

A modular analysis of the auxin signalling network Supplementary Figures

E. Farcot, C. Lavedrine, T. Vernoux

The parameters α_{XY} were varied over 5 orders of magnitude. By default, from comparable cases found in the literature they took values (in $nM^{-1}.min^{-1}$ for protein-protein association and min^{-1} for protein-promoter association) in the sample set

$$S_\alpha = \{0.001, 0.01, 0.1, 1.0, 10.0\},$$

whereas the parameters θ_{XY} were varied over 6 orders of magnitude, taking default values (in min^{-1} for protein-protein association and $nM.min^{-1}$ for protein-promoter association) in

$$S_\theta = \{0, 0.001, 0.01, 0.1, 1.0, 10.0\}.$$

The value 0 was not included for α_{XY} because it corresponds to an extreme situation where no dimers can form, which leads to an absence of response to any auxin input. Even with the assumptions made at this point, exploring all combinations involves 900 cases, which would have led to an unreasonably long computational time, approaching the time it would take to perform actual wet lab experiments. We thus restricted the exploration by imposing that protein-protein and protein-promoter constants vary by at most one order of magnitude.

We use a notational convention to describe all the considered cases. Denote $\alpha_P < \alpha_{AG}$ a situation where the association constants for protein-promoter vary in S_α and association constants are one order of magnitude smaller, hence varying in $S_\alpha/10 = \{0.0001, 0.001, 0.01, 0.1, 1.0\}$. Similarly, we denote $\theta_P < \theta_{AG}$ when the dissociation constants for protein-protein binding reaction are one order of magnitude smaller than constants for protein-promoter dissociation (and thus vary in $S_\theta/10$ instead of S_θ). The table below summarizes all the cases we have explored, with the natural generalization of the notation just described:

	$\alpha_P = \alpha_{AG}$	$\alpha_{AG} < \alpha_P$	$\alpha_P < \alpha_{AG}$
$\theta_P = \theta_{AG}$	"default"	A	B
$\theta_{AG} < \theta_P$	C	E	G
$\theta_P < \theta_{AG}$	D	F	H

The following 16 pages present the calculated landscapes for cases A, B, C, D, E, F, G and H, in this order. For each of the eight cases, the four outputs are represented on a grid of association/dissociation constants, followed by zooms near $\lambda = 0$ and $\lambda = 1$ of the landscapes for the relative and absolute responses.

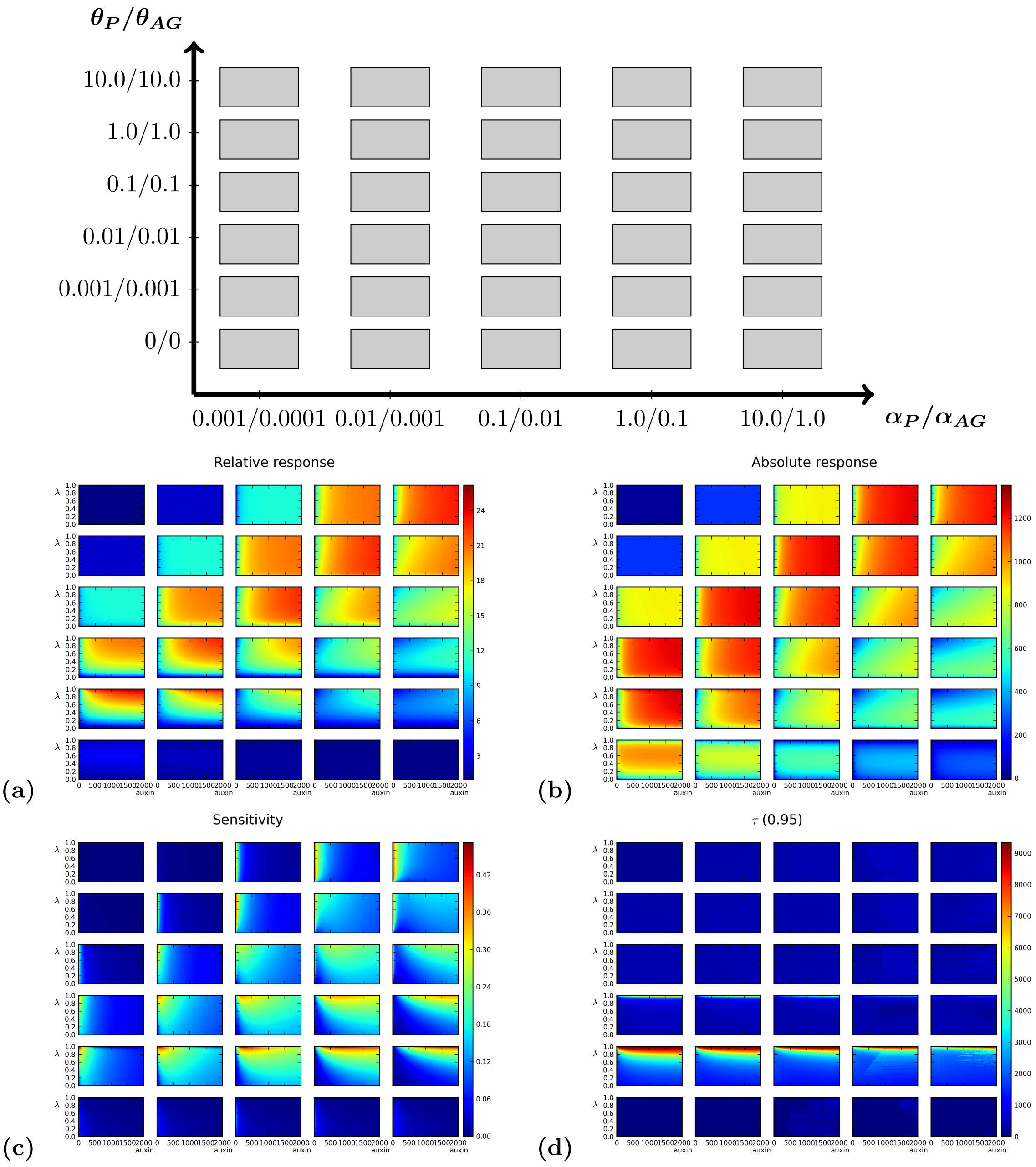

Figure 1. Case A. Output landscapes as functions of auxin level x (abscissae) and balance between the two core mechanisms, parametrized by λ (ordinates). The values of association (resp. dissociation) constants for protein-protein, α_P (resp. θ_P) and protein-promoter, α_{AG} (resp. θ_{AG}) are shown on top. **(a)** Relative response $\rho_{rel}(x, \lambda)$. **(b)** Absolute response $\rho_{abs}(x, \lambda)$. **(c)** Sensitivity $\sigma(x, \lambda)$. **(d)** Response time $\tau(x, \lambda)$. For each landscape (x, λ) span a 200×200 regular grid on the rectangle $[0, 2000] \times [0, 1]$.

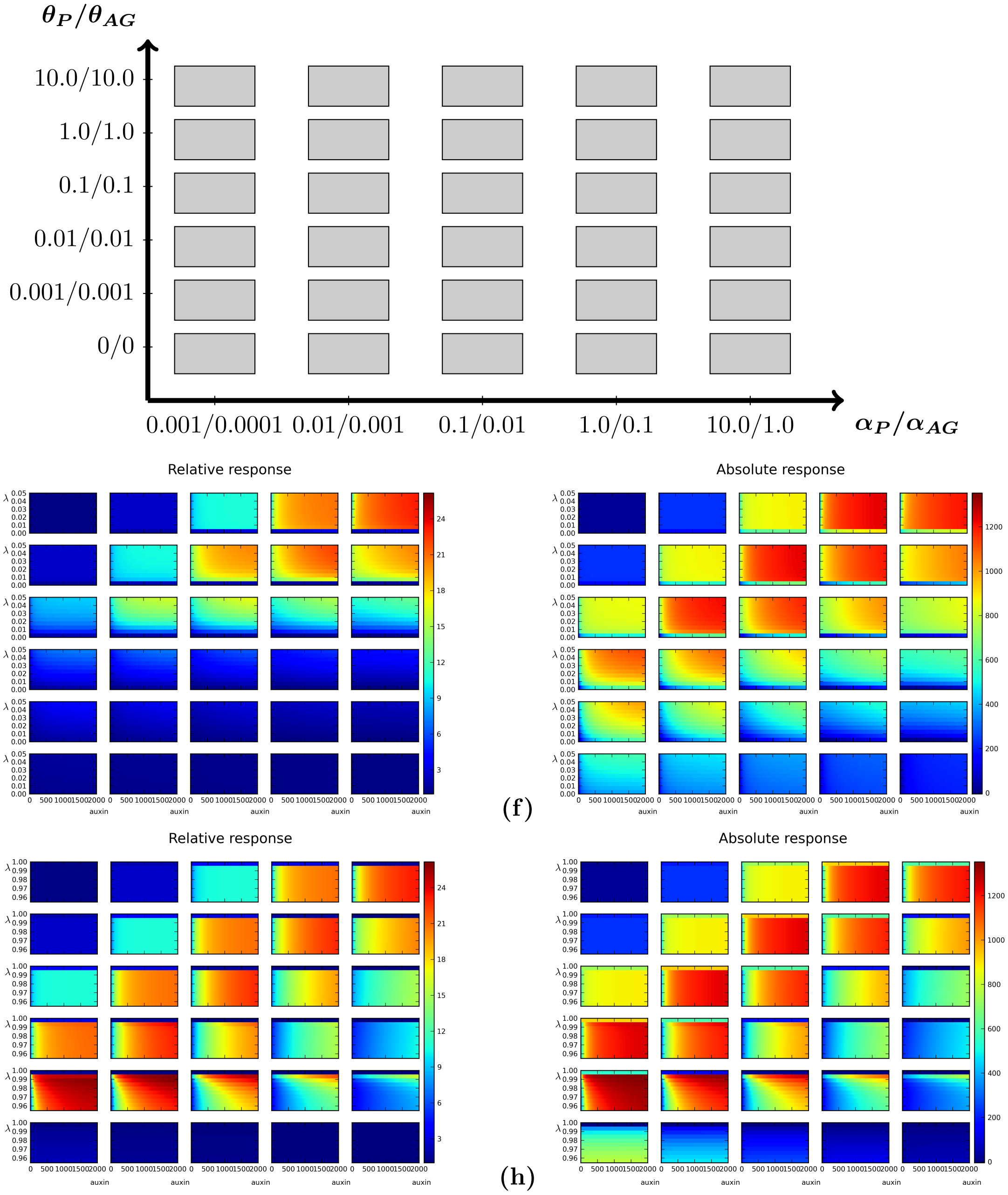

Figure 2. Case A. Output landscapes as functions of auxin level x (abscissae) and balance between the two core mechanisms, parametrized by λ (ordinates). The values of association (resp. dissociation) constants for protein-protein, α_P (resp. θ_P) and protein-promoter, α_{AG} (resp. θ_{AG}) are shown on top. **(e)** Zoom near $\lambda = 0$ of the relative response $\rho_{rel}(x, \lambda)$. **(f)** Zoom near $\lambda = 0$ of the absolute response $\rho_{abs}(x, \lambda)$. **(g)** Zoom near $\lambda = 1$ of the relative response $\rho_{rel}(x, \lambda)$. **(h)** Zoom near $\lambda = 1$ of the absolute response $\rho_{abs}(x, \lambda)$. For each landscape (x, λ) span a 200×200 regular grid on the rectangle $[0, 2000] \times [0, 1]$.

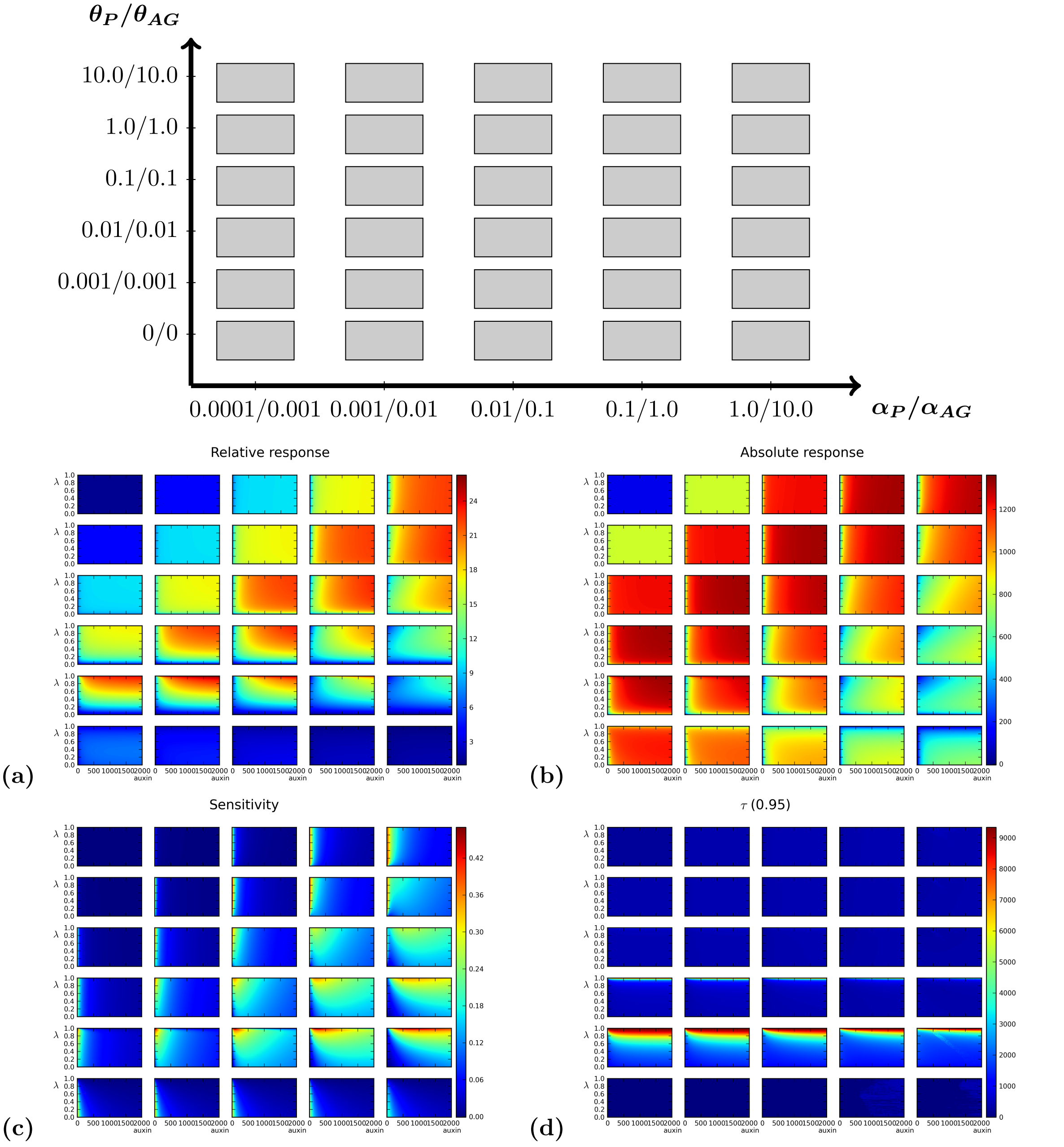

Figure 3. Case B. Output landscapes as functions of auxin level x (abscissae) and balance between the two core mechanisms, parametrized by λ (ordinates). The values of association (resp. dissociation) constants for protein-protein, α_P (resp. θ_P) and protein-promoter, α_{AG} (resp. θ_{AG}) are shown on top. **(a)** Relative response $\rho_{rel}(x, \lambda)$. **(b)** Absolute response $\rho_{abs}(x, \lambda)$. **(c)** Sensitivity $\sigma(x, \lambda)$. **(d)** Response time $\tau(x, \lambda)$. For each landscape (x, λ) span a 200×200 regular grid on the rectangle $[0, 2000] \times [0, 1]$.

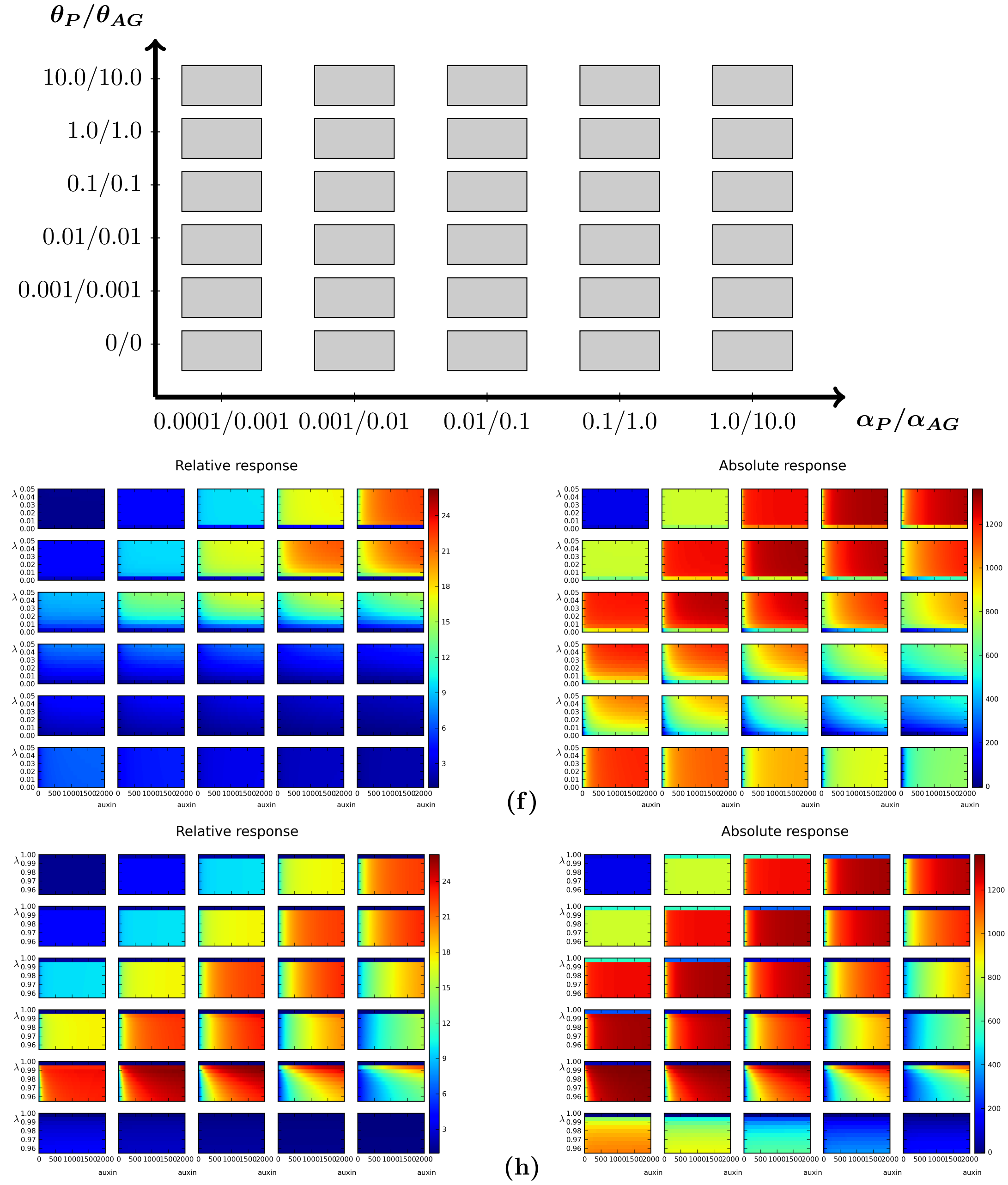

Figure 4. Case B. Output landscapes as functions of auxin level x (abscissae) and balance between the two core mechanisms, parametrized by λ (ordinates). The values of association (resp. dissociation) constants for protein-protein, α_P (resp. θ_P) and protein-promoter, α_{AG} (resp. θ_{AG}) are shown on top. **(e)** Zoom near $\lambda = 0$ of the relative response $\rho_{rel}(x, \lambda)$. **(f)** Zoom near $\lambda = 0$ of the absolute response $\rho_{abs}(x, \lambda)$. **(g)** Zoom near $\lambda = 1$ of the relative response $\rho_{rel}(x, \lambda)$. **(h)** Zoom near $\lambda = 1$ of the absolute response $\rho_{abs}(x, \lambda)$. For each landscape (x, λ) span a 200×200 regular grid on the rectangle $[0, 2000] \times [0, 1]$.

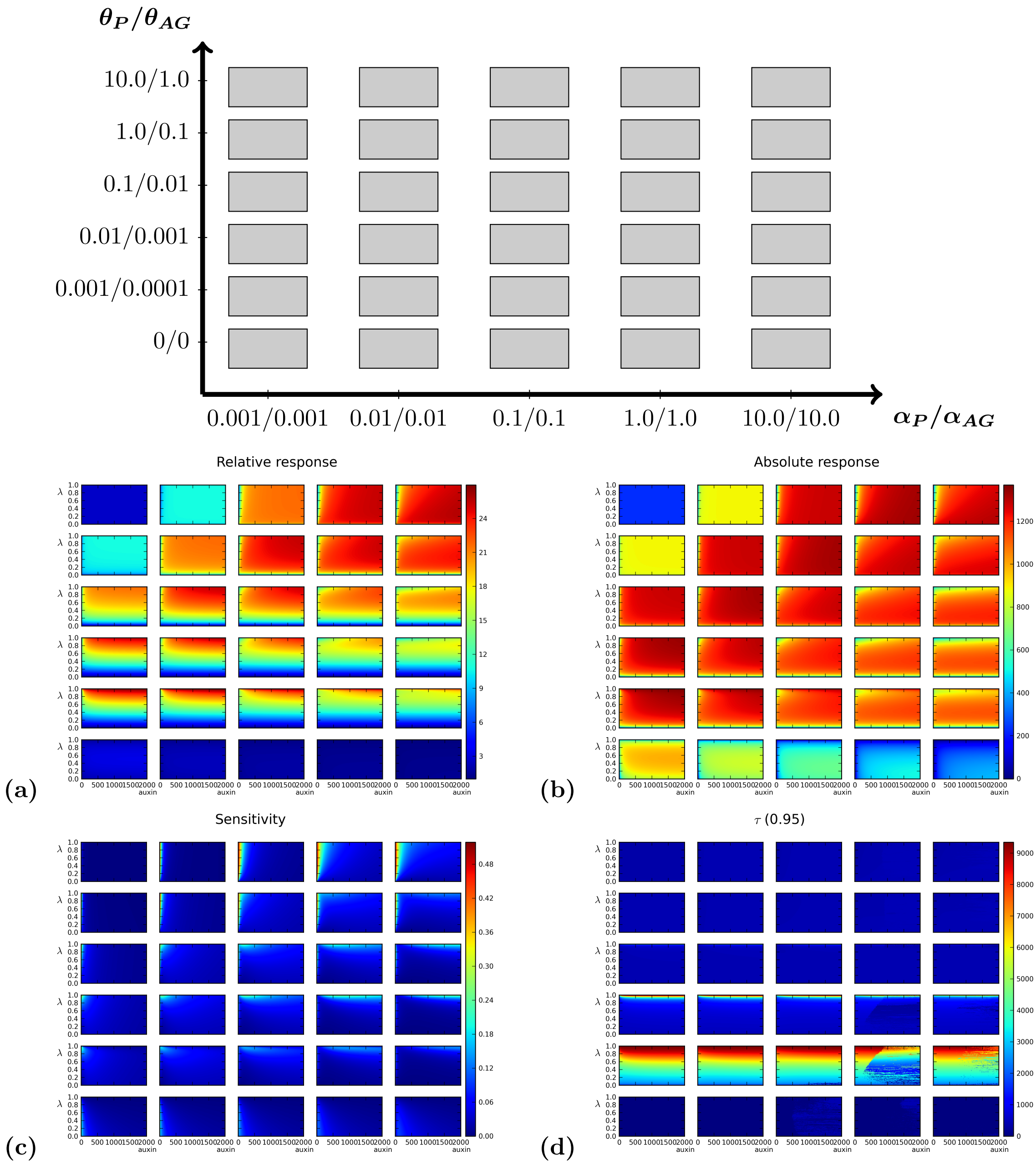

Figure 5. Case C. Output landscapes as functions of auxin level x (abscissae) and balance between the two core mechanisms, parametrized by λ (ordinates). The values of association (resp. dissociation) constants for protein-protein, α_P (resp. θ_P) and protein-promoter, α_{AG} (resp. θ_{AG}) are shown on top. **(a)** Relative response $\rho_{rel}(x, \lambda)$. **(b)** Absolute response $\rho_{abs}(x, \lambda)$. **(c)** Sensitivity $\sigma(x, \lambda)$. **(d)** Response time $\tau(x, \lambda)$. For each landscape (x, λ) span a 200×200 regular grid on the rectangle $[0, 2000] \times [0, 1]$.

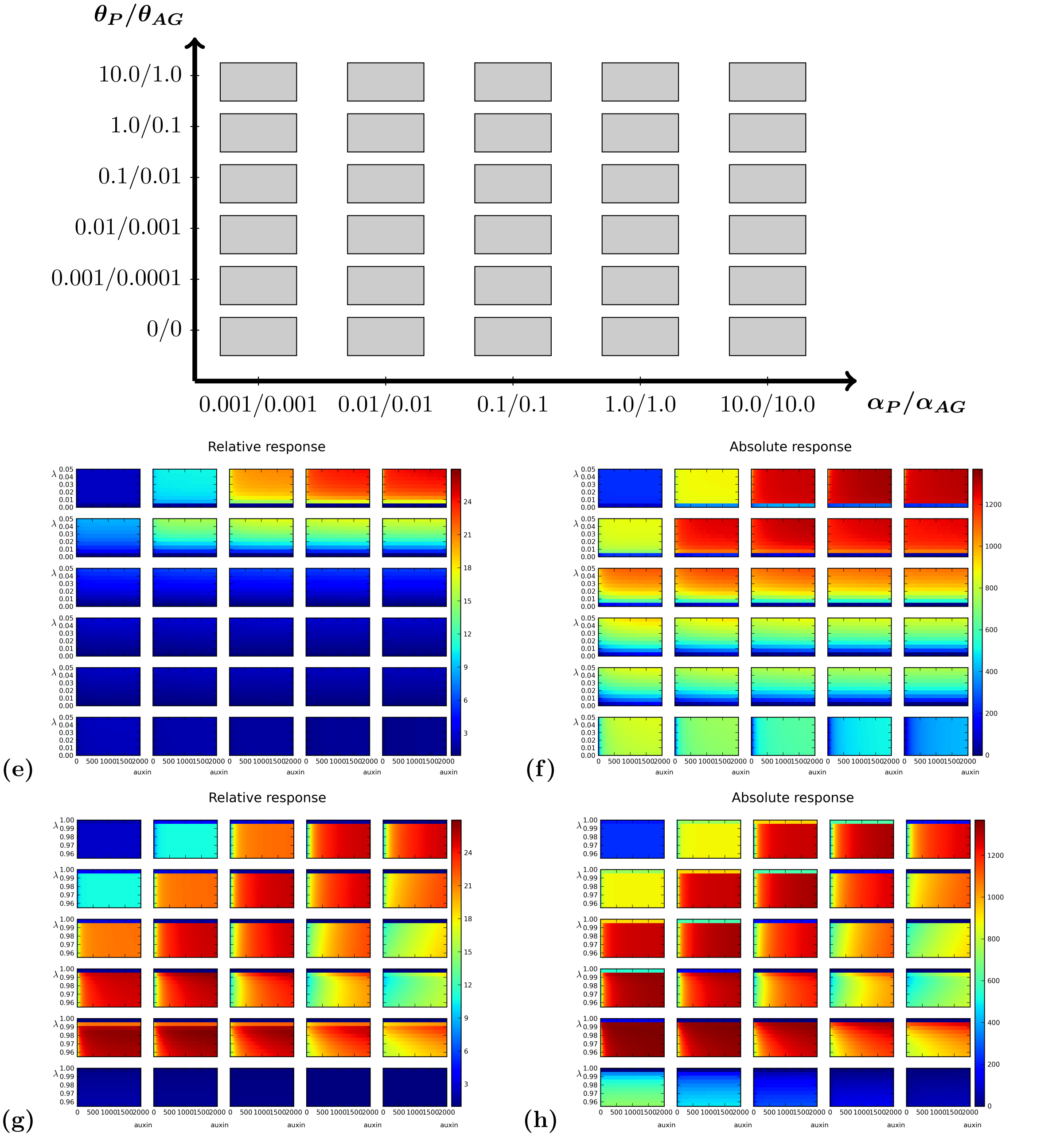

Figure 6. Case C. Output landscapes as functions of auxin level x (abscissae) and balance between the two core mechanisms, parametrized by λ (ordinates). The values of association (resp. dissociation) constants for protein-protein, α_P (resp. θ_P) and protein-promoter, α_{AG} (resp. θ_{AG}) are shown on top. **(e)** Zoom near $\lambda = 0$ of the relative response $\rho_{rel}(x, \lambda)$. **(f)** Zoom near $\lambda = 0$ of the absolute response $\rho_{abs}(x, \lambda)$. **(g)** Zoom near $\lambda = 1$ of the relative response $\rho_{rel}(x, \lambda)$. **(h)** Zoom near $\lambda = 1$ of the absolute response $\rho_{abs}(x, \lambda)$. For each landscape (x, λ) span a 200×200 regular grid on the rectangle $[0, 2000] \times [0, 1]$.

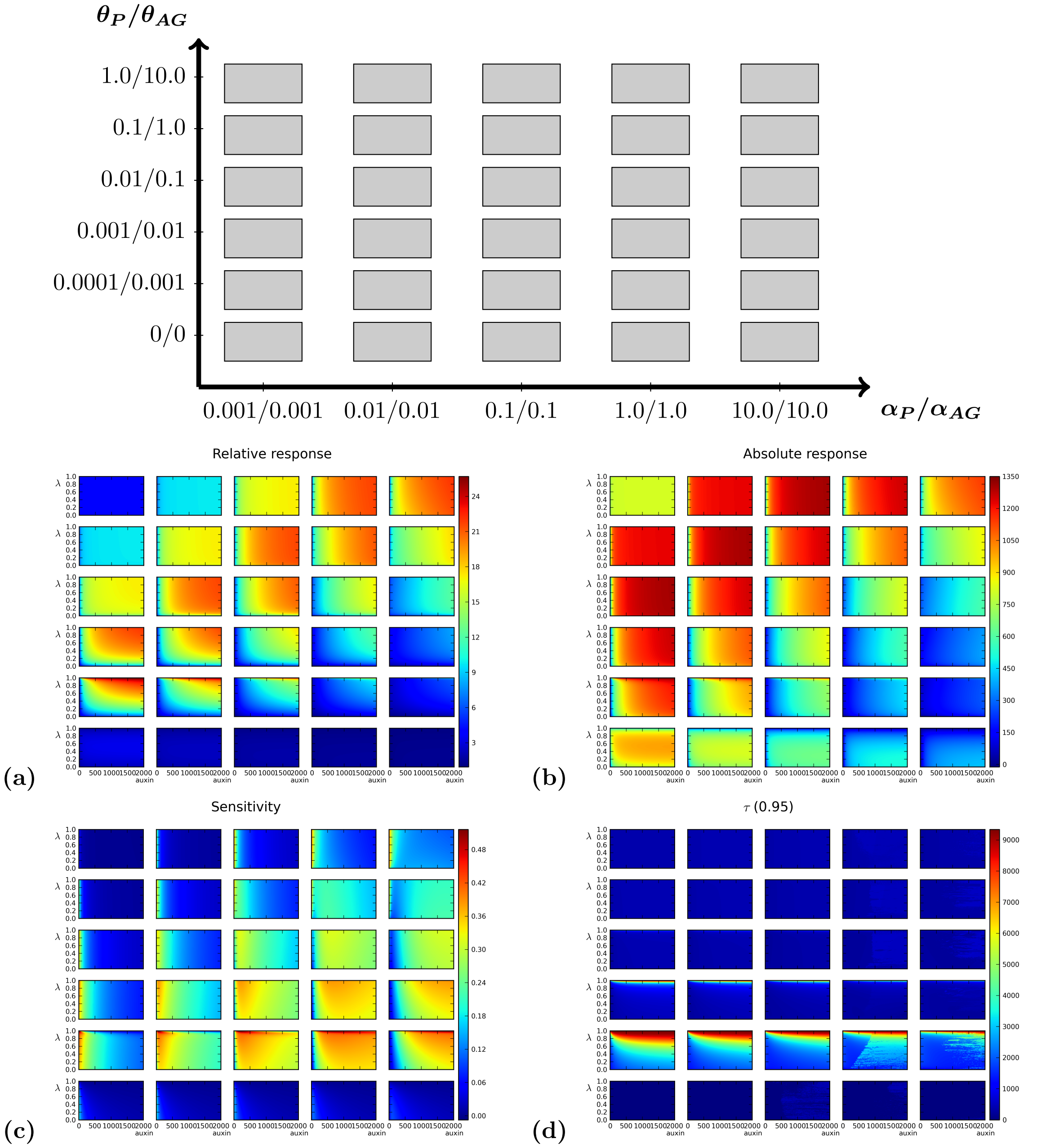

Figure 7. Case D. Output landscapes as functions of auxin level x (abscissae) and balance between the two core mechanisms, parametrized by λ (ordinates). The values of association (resp. dissociation) constants for protein-protein, α_P (resp. θ_P) and protein-promoter, α_{AG} (resp. θ_{AG}) are shown on top. **(a)** Relative response $\rho_{rel}(x, \lambda)$. **(b)** Absolute response $\rho_{abs}(x, \lambda)$. **(c)** Sensitivity $\sigma(x, \lambda)$. **(d)** Response time $\tau(x, \lambda)$. For each landscape (x, λ) span a 200×200 regular grid on the rectangle $[0, 2000] \times [0, 1]$.

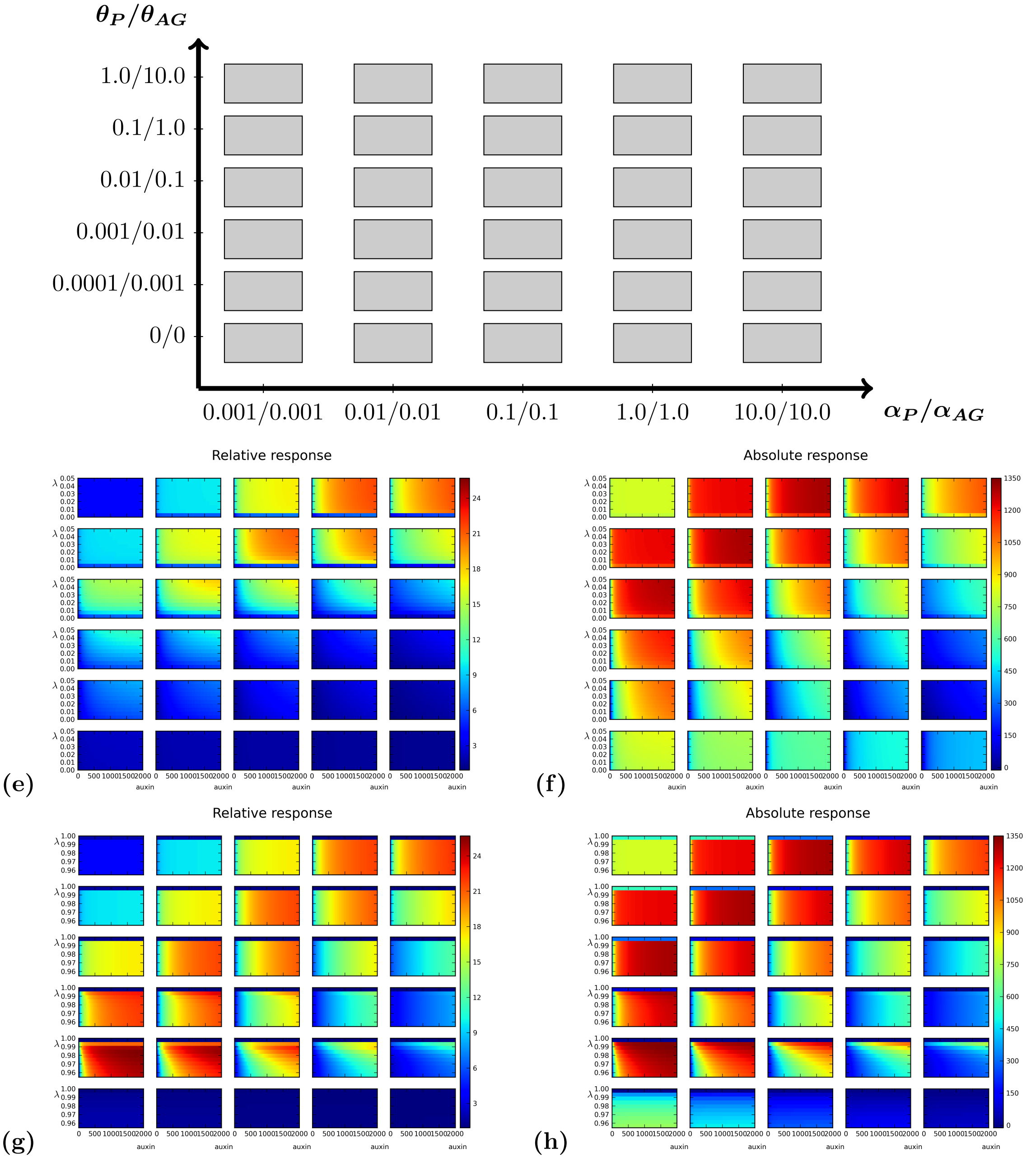

Figure 8. Case D. Output landscapes as functions of auxin level x (abscissae) and balance between the two core mechanisms, parametrized by λ (ordinates). The values of association (resp. dissociation) constants for protein-protein, α_P (resp. θ_P) and protein-promoter, α_{AG} (resp. θ_{AG}) are shown on top. **(e)** Zoom near $\lambda = 0$ of the relative response $\rho_{rel}(x, \lambda)$. **(f)** Zoom near $\lambda = 0$ of the absolute response $\rho_{abs}(x, \lambda)$. **(g)** Zoom near $\lambda = 1$ of the relative response $\rho_{rel}(x, \lambda)$. **(h)** Zoom near $\lambda = 1$ of the absolute response $\rho_{abs}(x, \lambda)$. For each landscape (x, λ) span a 200×200 regular grid on the rectangle $[0, 2000] \times [0, 1]$.

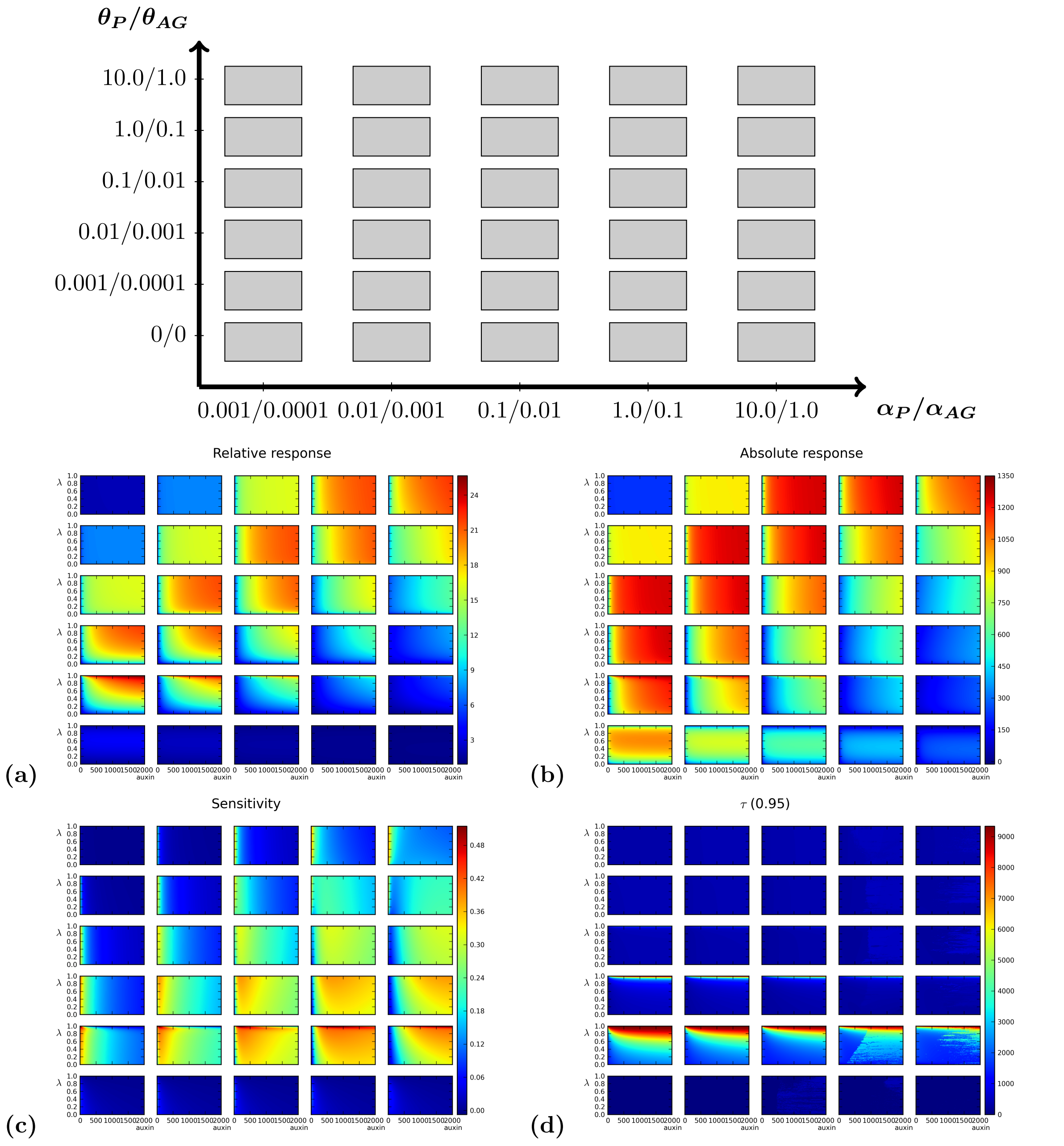

Figure 9. Case E. Output landscapes as functions of auxin level x (abscissae) and balance between the two core mechanisms, parametrized by λ (ordinates). The values of association (resp. dissociation) constants for protein-protein, α_P (resp. θ_P) and protein-promoter, α_{AG} (resp. θ_{AG}) are shown on top. **(a)** Relative response $\rho_{rel}(x, \lambda)$. **(b)** Absolute response $\rho_{abs}(x, \lambda)$. **(c)** Sensitivity $\sigma(x, \lambda)$. **(d)** Response time $\tau(x, \lambda)$. For each landscape (x, λ) span a 200×200 regular grid on the rectangle $[0, 2000] \times [0, 1]$.

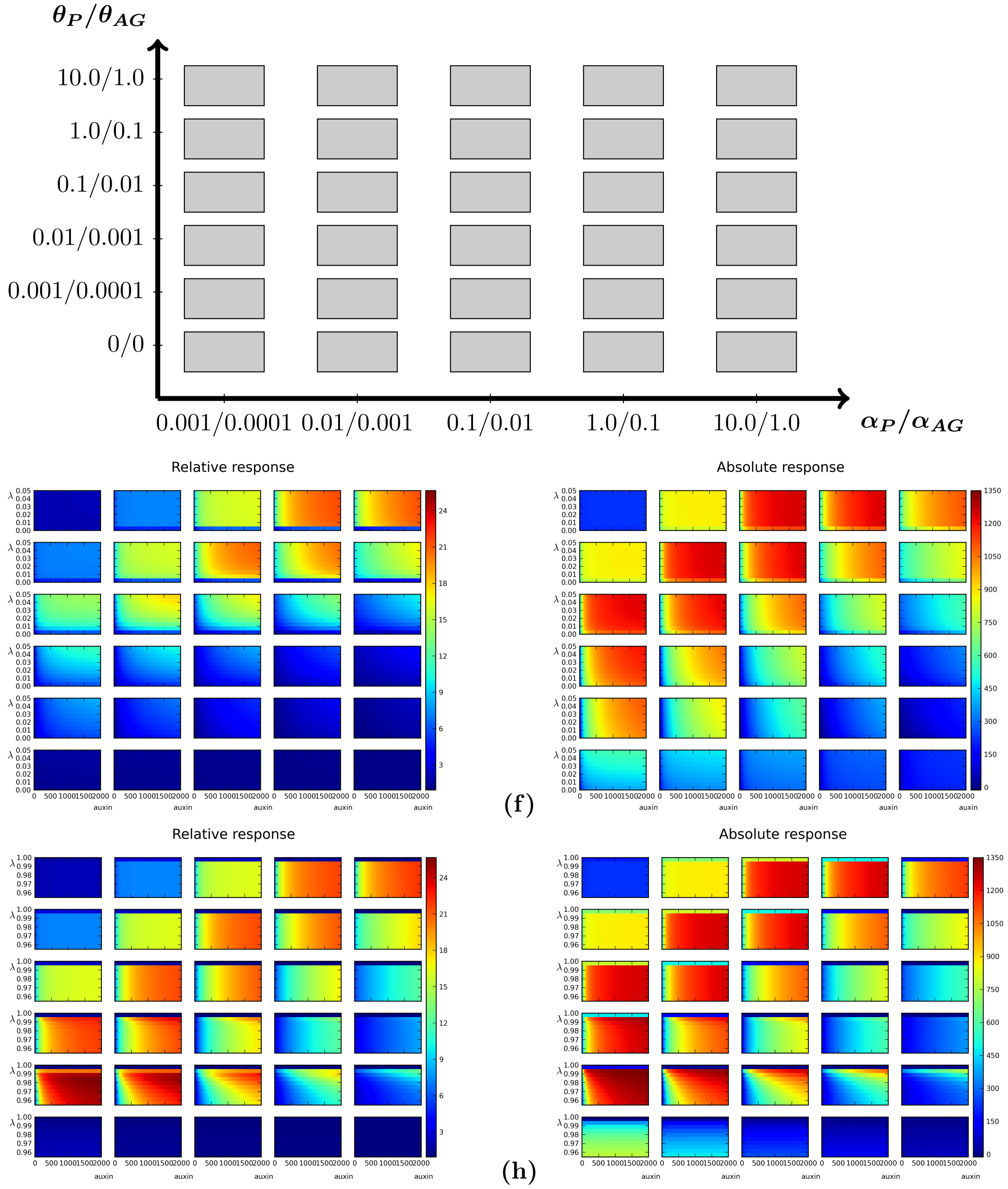

Figure 10. Case E. Output landscapes as functions of auxin level x (abscissae) and balance between the two core mechanisms, parametrized by λ (ordinates). The values of association (resp. dissociation) constants for protein-protein, α_P (resp. θ_P) and protein-promoter, α_{AG} (resp. θ_{AG}) are shown on top. **(e)** Zoom near $\lambda = 0$ of the relative response $\rho_{rel}(x, \lambda)$. **(f)** Zoom near $\lambda = 0$ of the absolute response $\rho_{abs}(x, \lambda)$. **(g)** Zoom near $\lambda = 1$ of the relative response $\rho_{rel}(x, \lambda)$. **(h)** Zoom near $\lambda = 1$ of the absolute response $\rho_{abs}(x, \lambda)$. For each landscape (x, λ) span a 200×200 regular grid on the rectangle $[0, 2000] \times [0, 1]$.

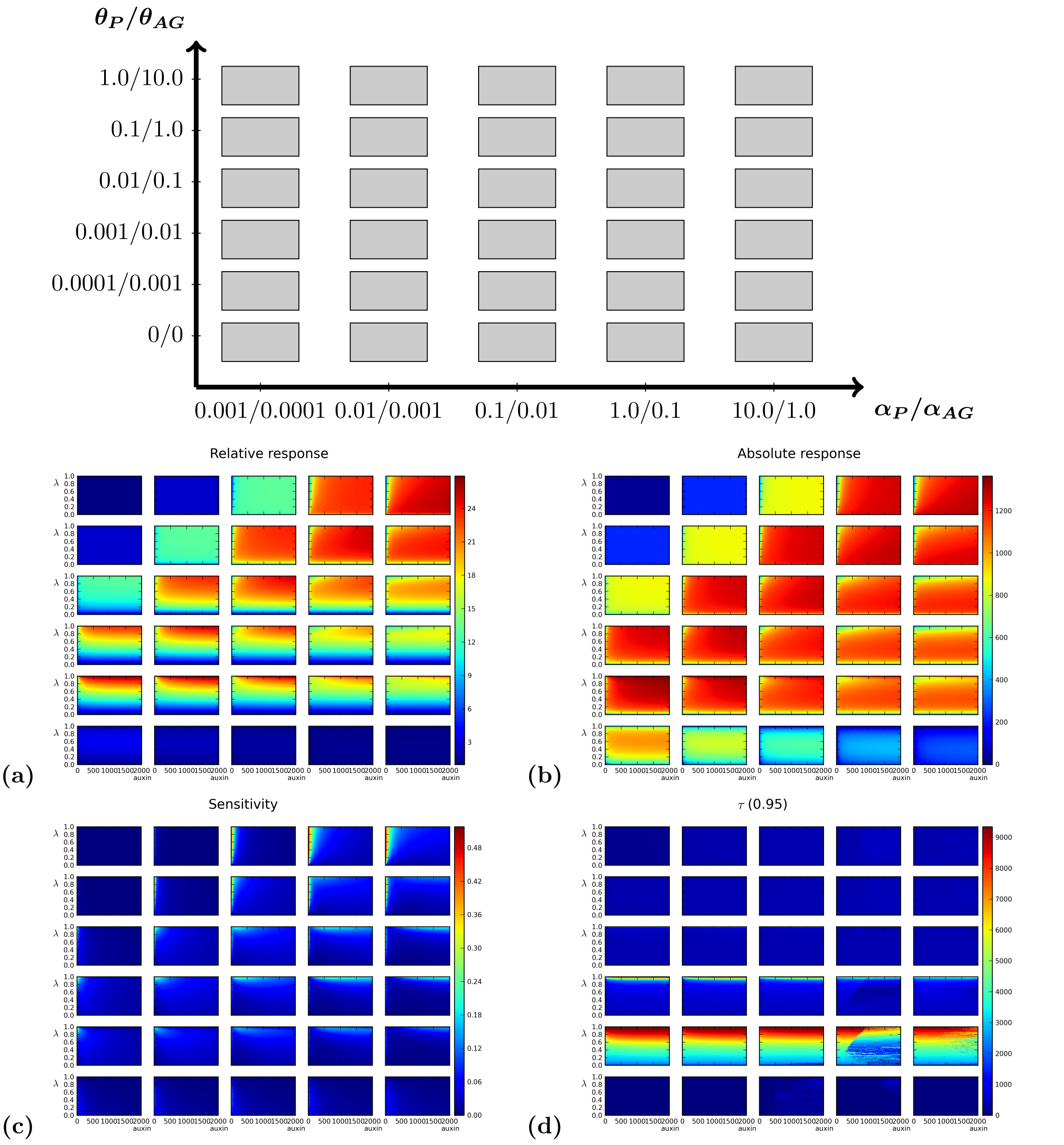

Figure 11. Case F. Output landscapes as functions of auxin level x (abscissae) and balance between the two core mechanisms, parametrized by λ (ordinates). The values of association (resp. dissociation) constants for protein-protein, α_P (resp. θ_P) and protein-promoter, α_{AG} (resp. θ_{AG}) are shown on top. **(a)** Relative response $\rho_{rel}(x, \lambda)$. **(b)** Absolute response $\rho_{abs}(x, \lambda)$. **(c)** Sensitivity $\sigma(x, \lambda)$. **(d)** Response time $\tau(x, \lambda)$. For each landscape (x, λ) span a 200×200 regular grid on the rectangle $[0, 2000] \times [0, 1]$.

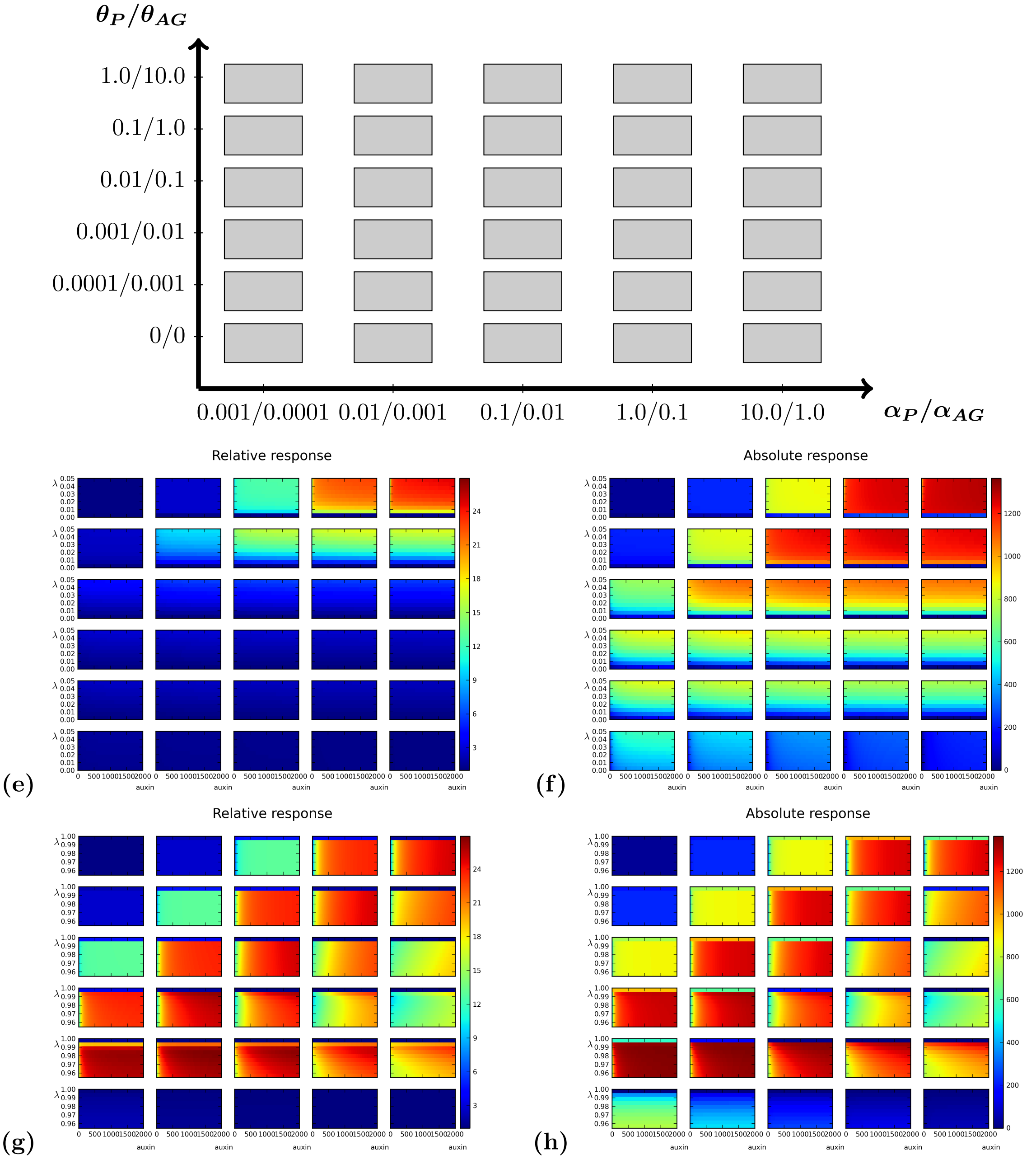

Figure 12. Case F. Output landscapes as functions of auxin level x (abscissae) and balance between the two core mechanisms, parametrized by λ (ordinates). The values of association (resp. dissociation) constants for protein-protein, α_P (resp. θ_P) and protein-promoter, α_{AG} (resp. θ_{AG}) are shown on top. **(e)** Zoom near $\lambda = 0$ of the relative response $\rho_{rel}(x, \lambda)$. **(f)** Zoom near $\lambda = 0$ of the absolute response $\rho_{abs}(x, \lambda)$. **(g)** Zoom near $\lambda = 1$ of the relative response $\rho_{rel}(x, \lambda)$. **(h)** Zoom near $\lambda = 1$ of the absolute response $\rho_{abs}(x, \lambda)$. For each landscape (x, λ) span a 200×200 regular grid on the rectangle $[0, 2000] \times [0, 1]$.

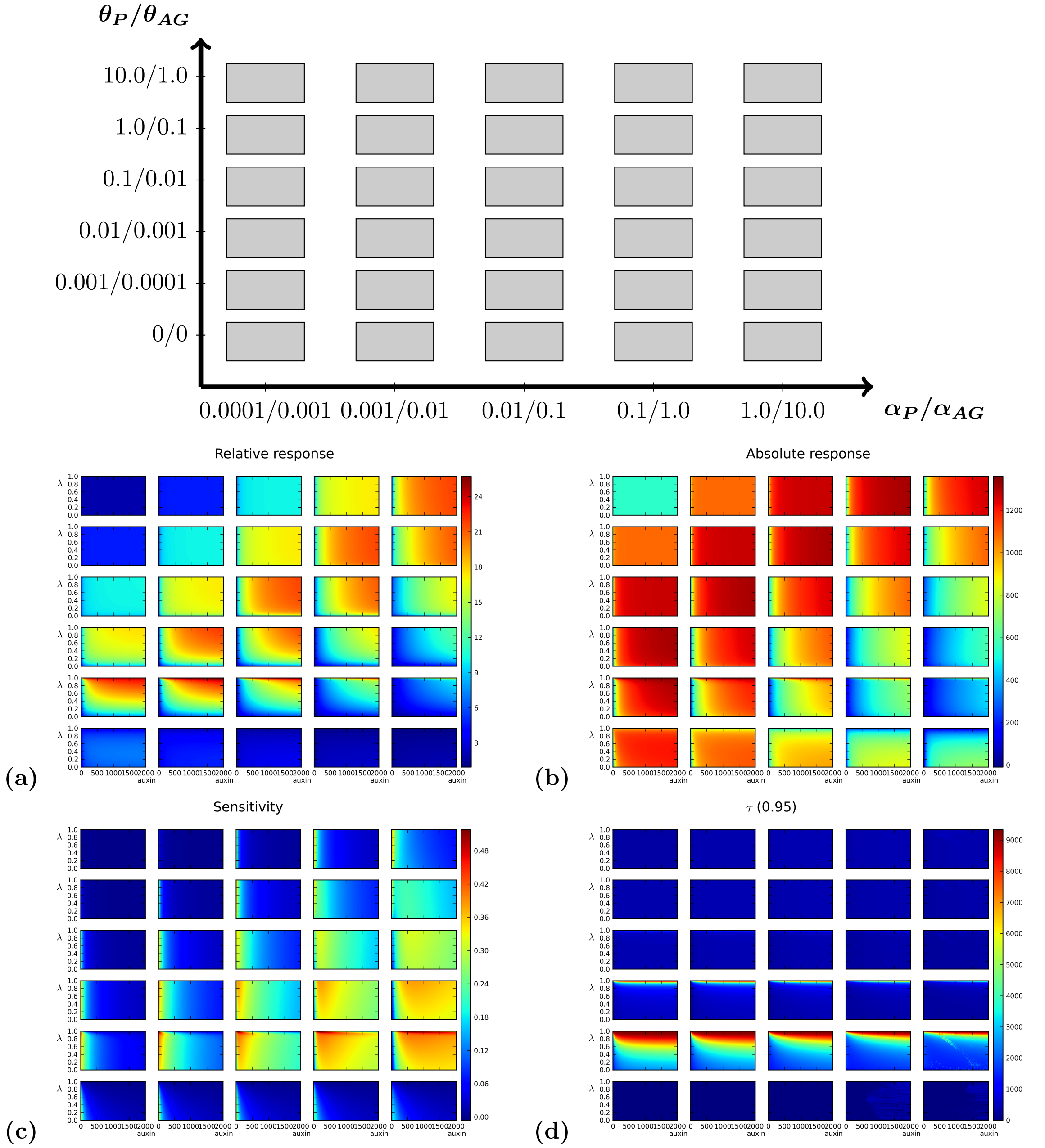

Figure 13. Case G. Output landscapes as functions of auxin level x (abscissae) and balance between the two core mechanisms, parametrized by λ (ordinates). The values of association (resp. dissociation) constants for protein-protein, α_P (resp. θ_P) and protein-promoter, α_{AG} (resp. θ_{AG}) are shown on top. **(a)** Relative response $\rho_{rel}(x, \lambda)$. **(b)** Absolute response $\rho_{abs}(x, \lambda)$. **(c)** Sensitivity $\sigma(x, \lambda)$. **(d)** Response time $\tau(x, \lambda)$. For each landscape (x, λ) span a 200×200 regular grid on the rectangle $[0, 2000] \times [0, 1]$.

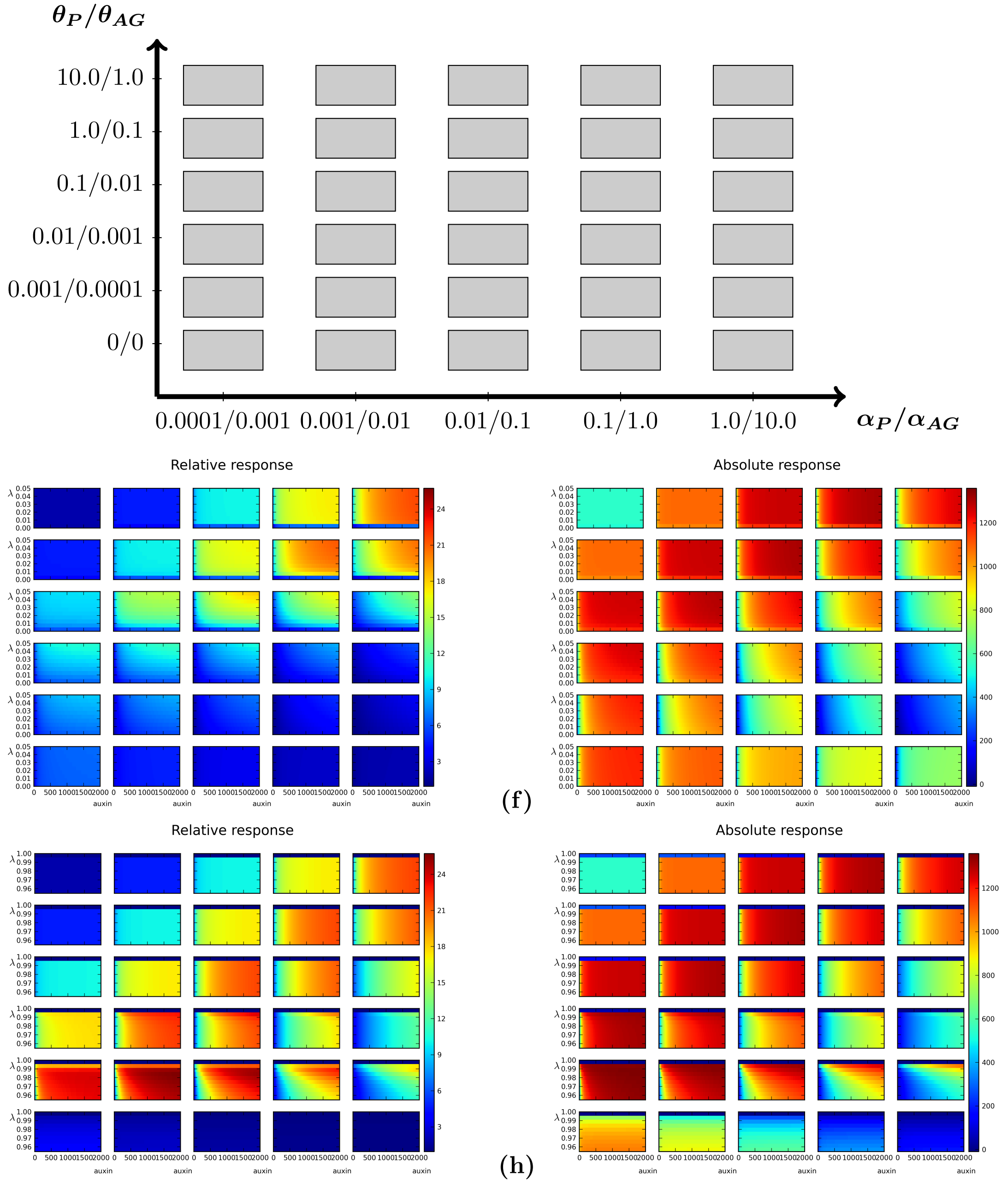

Figure 14. Case G. Output landscapes as functions of auxin level x (abscissae) and balance between the two core mechanisms, parametrized by λ (ordinates). The values of association (resp. dissociation) constants for protein-protein, α_P (resp. θ_P) and protein-promoter, α_{AG} (resp. θ_{AG}) are shown on top. **(e)** Zoom near $\lambda = 0$ of the relative response $\rho_{rel}(x, \lambda)$. **(f)** Zoom near $\lambda = 0$ of the absolute response $\rho_{abs}(x, \lambda)$. **(g)** Zoom near $\lambda = 1$ of the relative response $\rho_{rel}(x, \lambda)$. **(h)** Zoom near $\lambda = 1$ of the absolute response $\rho_{abs}(x, \lambda)$. For each landscape (x, λ) span a 200×200 regular grid on the rectangle $[0, 2000] \times [0, 1]$.

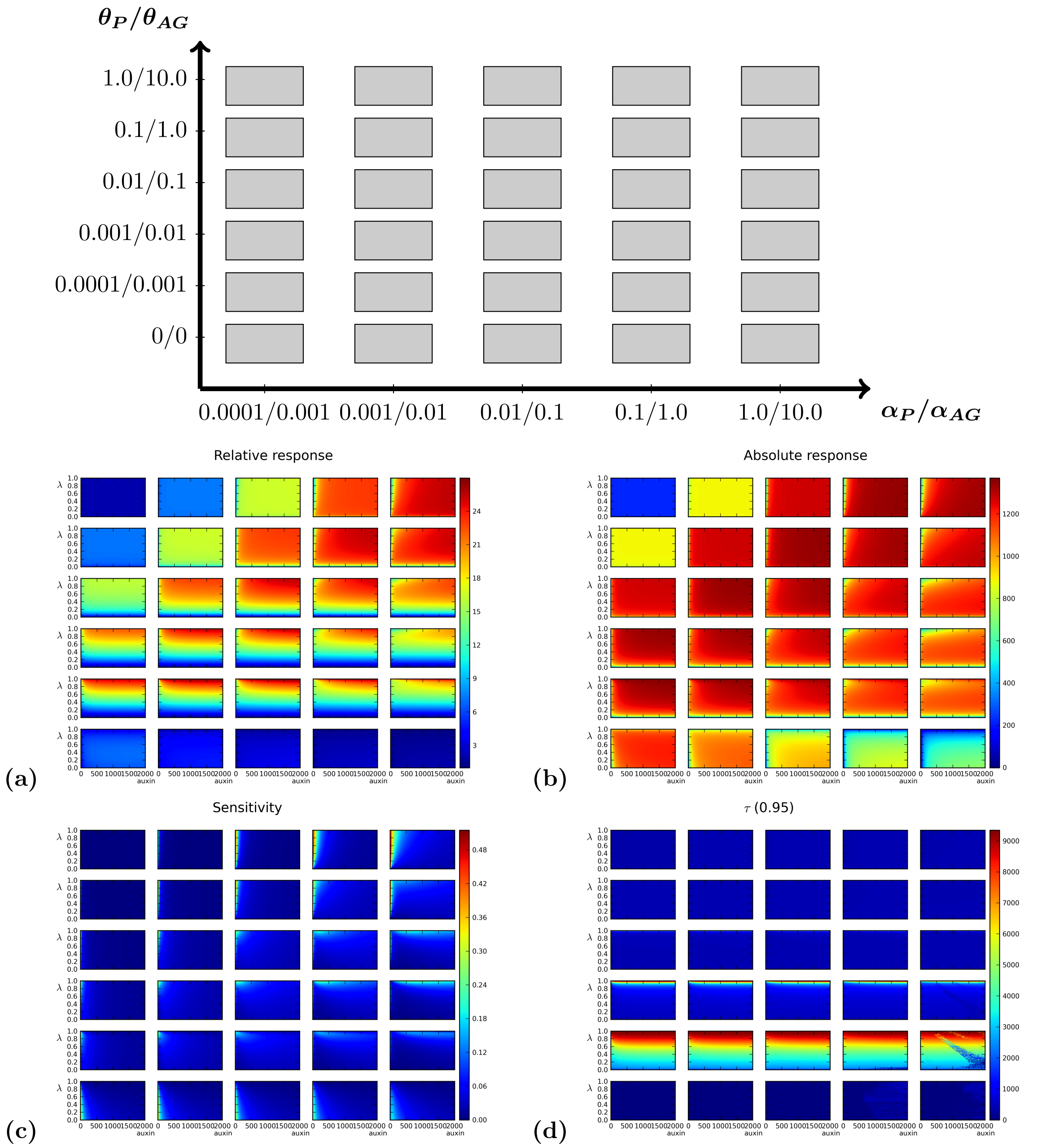

Figure 15. Case H. Output landscapes as functions of auxin level x (abscissae) and balance between the two core mechanisms, parametrized by λ (ordinates). The values of association (resp. dissociation) constants for protein-protein, α_P (resp. θ_P) and protein-promoter, α_{AG} (resp. θ_{AG}) are shown on top. **(a)** Relative response $\rho_{rel}(x, \lambda)$. **(b)** Absolute response $\rho_{abs}(x, \lambda)$. **(c)** Sensitivity $\sigma(x, \lambda)$. **(d)** Response time $\tau(x, \lambda)$. For each landscape (x, λ) span a 200×200 regular grid on the rectangle $[0, 2000] \times [0, 1]$.

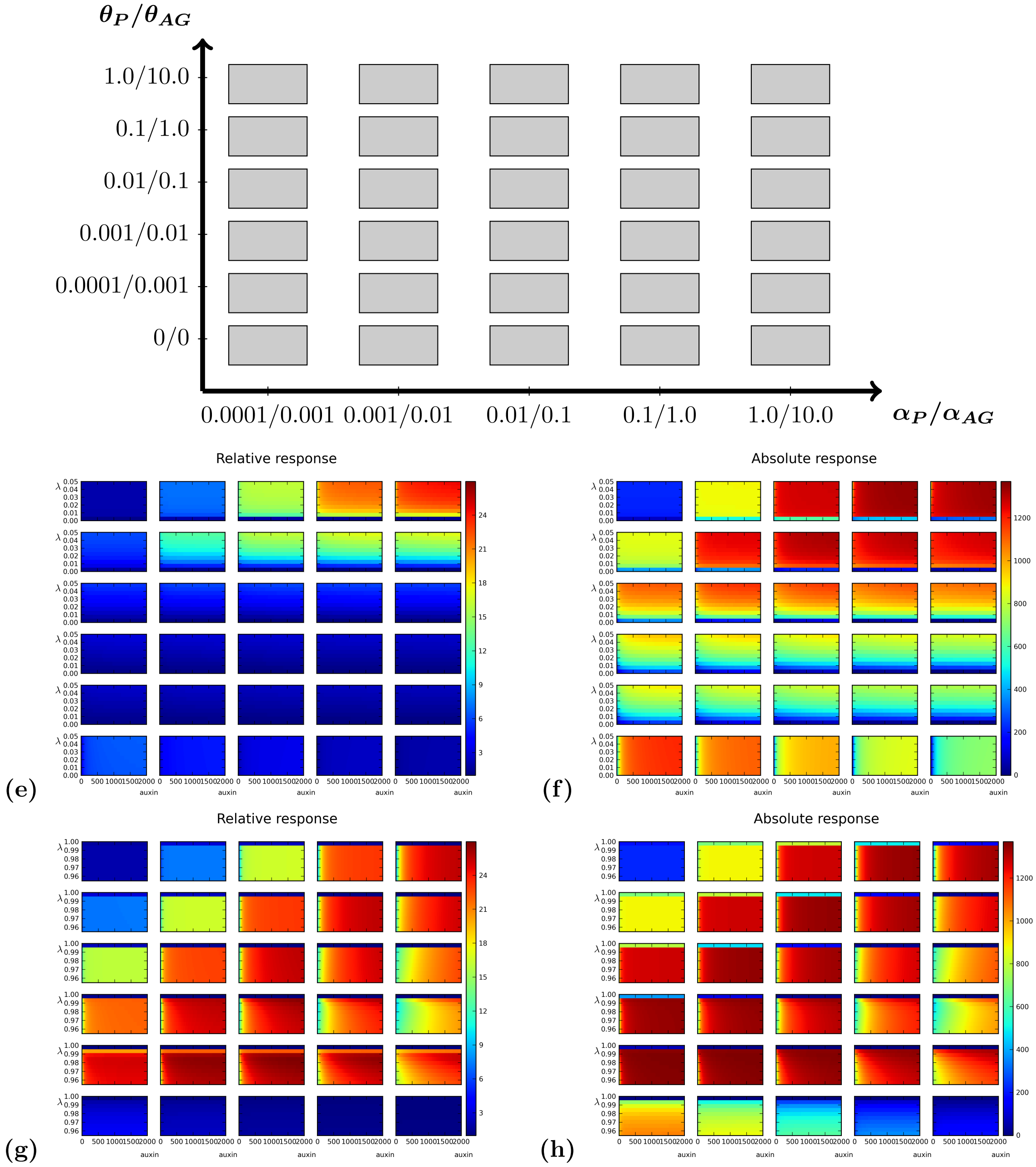

Figure 16. Case H. Output landscapes as functions of auxin level x (abscissae) and balance between the two core mechanisms, parametrized by λ (ordinates). The values of association (resp. dissociation) constants for protein-protein, α_P (resp. θ_P) and protein-promoter, α_{AG} (resp. θ_{AG}) are shown on top. **(e)** Zoom near $\lambda = 0$ of the relative response $\rho_{rel}(x, \lambda)$. **(f)** Zoom near $\lambda = 0$ of the absolute response $\rho_{abs}(x, \lambda)$. **(g)** Zoom near $\lambda = 1$ of the relative response $\rho_{rel}(x, \lambda)$. **(h)** Zoom near $\lambda = 1$ of the absolute response $\rho_{abs}(x, \lambda)$. For each landscape (x, λ) span a 200×200 regular grid on the rectangle $[0, 2000] \times [0, 1]$.
