# Supplementary material for: A Modular Analysis of the Auxin Signalling Network
Source: PLoS One. 2015 Mar 25;10(3):e0122231. doi: 10.1371/journal.pone.0122231 (PMC4373724; doi:10.1371/journal.pone.0122231)
Supplement: S2 Additional Information — (PDF) [file pone.0122231.s003.pdf]

A modular analysis of the auxin signalling network
Supplementary Figures: Alternative Models of Auxin Induced IAA Degradation

E. Farcot, C. Lavedrine, T. Vernoux

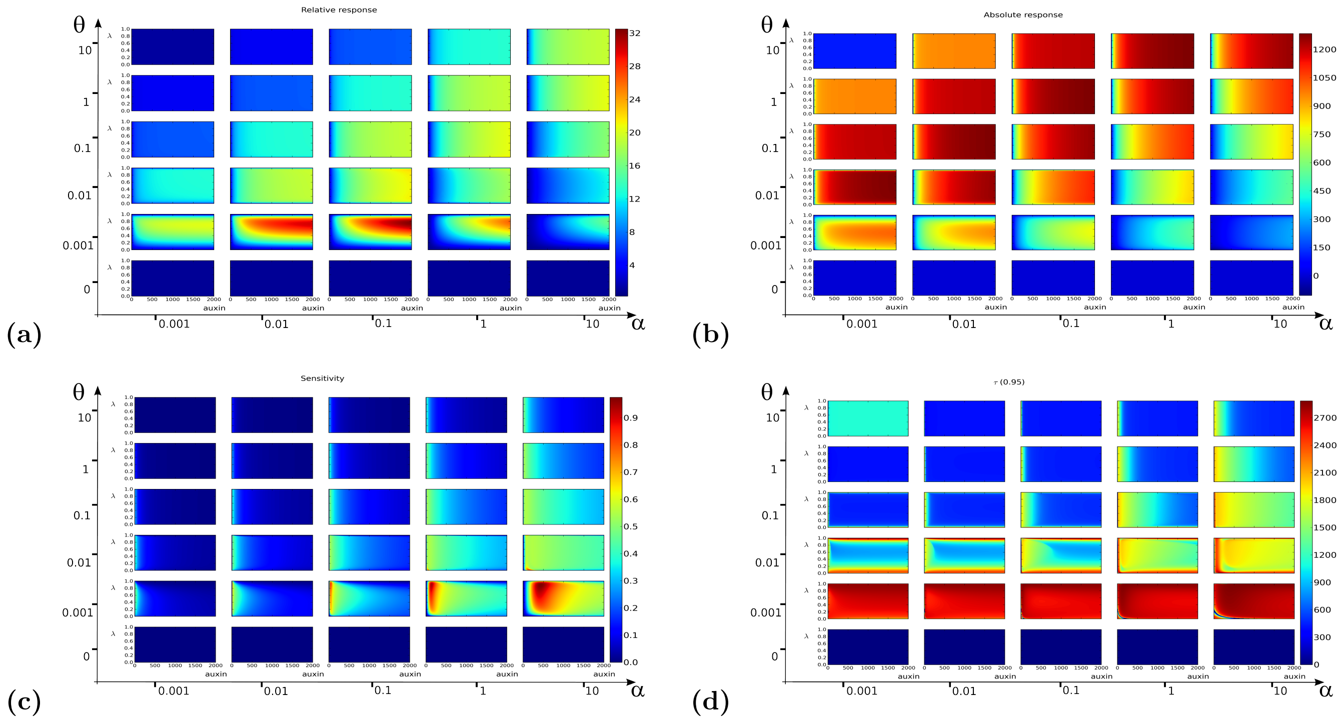

Figure 1. Model (M1). Output landscapes as functions of auxin level x (abscissae) and balance between the two core mechanisms, parametrized by λ (ordinates). (a) Relative response $\rho_{rel}(x, \lambda)$. **(b)** Absolute response $\rho_{abs}(x, \lambda)$. **(c)** Sensitivity $\sigma(x, \lambda)$. **(d)** Response time $\tau(x, \lambda)$. For each landscape (x, λ) span a 200×200 regular grid on the rectangle $[0, 2000] \times [0, 1]$.

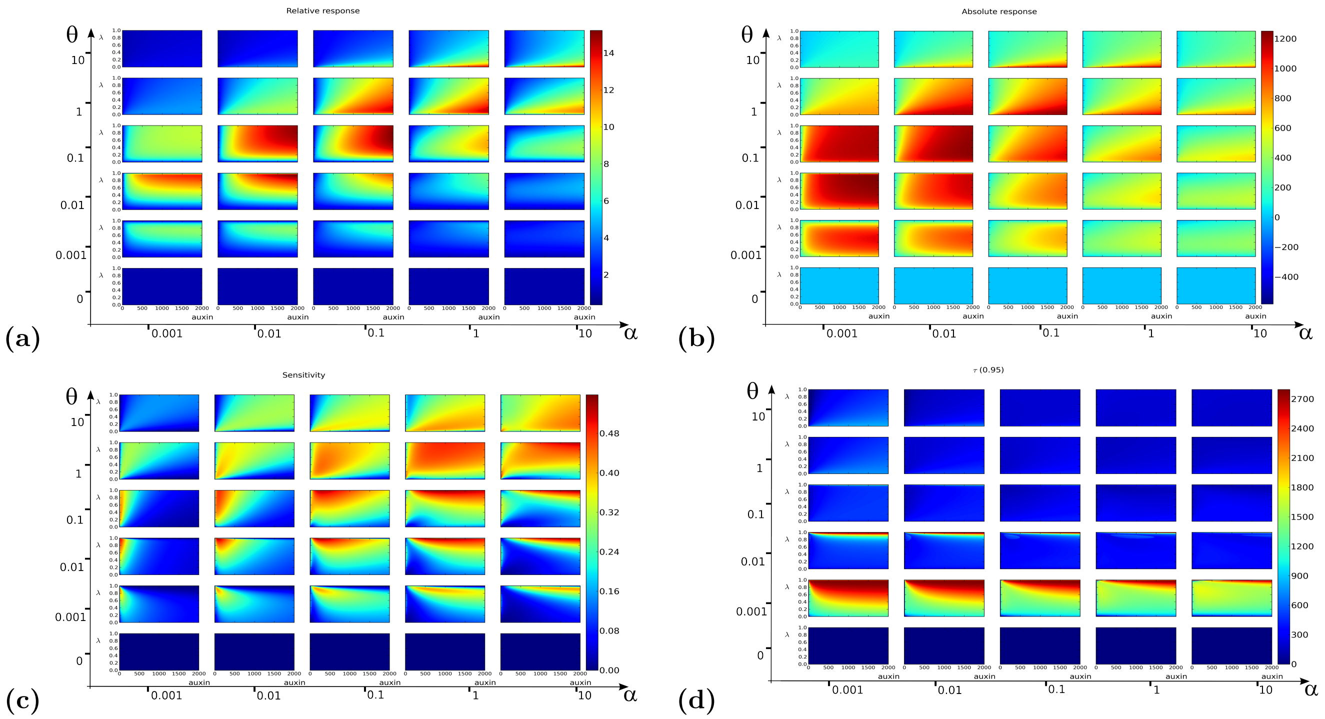

Figure 2. Model (M2). Output landscapes as functions of auxin level x (abscissae) and balance between the two core mechanisms, parametrized by λ (ordinates). (a) Relative response $\rho_{rel}(x,\lambda)$. **(b)** Absolute response $\rho_{abs}(x,\lambda)$. **(c)** Sensitivity $\sigma(x,\lambda)$. **(d)** Response time $\tau(x,\lambda)$. For each landscape (x,λ) span a 200×200 regular grid on the rectangle $[0,2000]\times[0,1]$.
